# Cartilage‐Penetrating Framework Nucleic Acid Nanoparticles Ameliorate Osteoarthritis by Promoting Drug Delivery and Chondrocyte Uptake

**DOI:** 10.1002/advs.202502661

**Published:** 2025-04-07

**Authors:** Kui Huang, Qiumei Li, Huixuan Lin, Qian Shen, Yaping Wu, Taoran Tian, Chuan Ma, Sirong Shi, Jingang Xiao, Yunfeng Lin

**Affiliations:** ^1^ Department of Oral and Maxillofacial Surgery The Affiliated Stomatological Hospital of Southwest Medical University Luzhou 646002 China; ^2^ State Key Laboratory of Oral Diseases National Clinical Research Center for Oral Diseases West China Hospital of Stomatology Sichuan University Chengdu 610041 China; ^3^ Luzhou Key Laboratory of Oral & Maxillofacial Reconstruction and Regeneration Luzhou 646600 China; ^4^ Department of Radiology The Affiliated Stomatological Hospital of Southwest Medical University Luzhou 646002 China; ^5^ Histology and Imaging Platform Core Facilities of West China Hospital Sichuan University Chengdu 610041 China; ^6^ Department of Oral and Maxillofacial Surgery The Affiliated Hospital of Southwest Medical University Luzhou 646000 China; ^7^ Department of Oral Implantology The Affiliated Stomatological Hospital of Southwest Medical University Luzhou 646002 China

**Keywords:** apoptosis, cartilage‐penetrating, chondrocyte uptake, framework nucleic acid nanoparticles, osteoarthritis, reactive oxygen species

## Abstract

Osteoarthritis (OA) is a chronic joint disease that causes a gradual deterioration of articular cartilage. A major challenge in OA treatment is the limited penetration and delivery efficiency of drugs to cartilage and chondrocytes due to the rapid clearance of drugs through synovial fluid in joints and the osmotic barrier of the cartilage extracellular matrix (ECM). To address this issue, a novel tetrahedral framework nucleic acid (tFNA)‐based nanomedicine delivery system (tFNA‐2WL) is first synthesized with excellent cartilage permeability and perfect chondrocyte endocytosis properties. After being loaded with ginsenoside Rb1 (Gin), the tFNA‐2WL&Gin complex not only penetrates the cartilage but also accumulates in the menisci, ligaments, and joint capsules, thus prolonging the residence time of Gin in OA rat knees. In vitro, tFNA‐2WL&Gin effectively promotes chondrogenesis, inhibits cartilage degradation by reducing apoptosis, and scavenges reactive oxygen species (ROS), outperforming free Gin. In OA rats, tFNA‐2WL&Gin restores gait, reduces osteophyte formation, inhibits synovial inflammation and hypertrophy, and protects cartilage from further damage more effectively than Gin and other nanomedicines. These results demonstrate the feasibility of tFNA‐2WL in improving the pharmacokinetics and efficacy of drugs and highlight the favorable curative effects of tFNA‐2WL&Gin for OA, offering a promising paradigm for translational medicine.

## Graphical abstract

Based on the poor cartilage penetration of tFNA, a novel tFNA‐2WL with excellent cartilage permeability and perfect chondrocyte endocytosis properties is designed and fabricated. As a result, tFNA‐2WL has a nearly seven‐fold increase in the penetration depth into the cartilage, with the same excellent cell entry properties as tFNA. After being loaded with ginsenoside Rb1 (Gin), the tFNA‐2WL&Gin excellently rebalances the imbalanced anabolic and catabolic metabolism of OA chondrocytes by reducing apoptosis and scavenging ROS and effectively ameliorates the progression of OA rats.

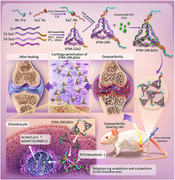

## 1 Introduction

Osteoarthritis (OA) is a prevalent degenerative joint disease affecting over 500 million individuals worldwide, with substantial social and economic impacts.^[^ ^1^ ^]^ It is primarily characterized by the deterioration of local articular cartilage, inflammation of knee joints, and pathological modifications of subchondral bone, ultimately leading to chronic pain and functional impairment of the affected joints.^[^ ^2^ ^]^ As one of the main pathologic features of OA, articular cartilage deterioration persists throughout the disease process, and numerous studies have demonstrated that articular cartilage degradation causes oxidative stress and cartilage dyshomeostasis.^[^ ^3^ ^]^ Chondrocytes, the only cells in articular cartilage, play a central role in maintaining articular cartilage homeostasis by producing anabolic factors such as aggrecan (ACAN) and type II collagen (Col II) and catabolic factors such as matrix metalloproteases (MMPs) and a disintegrin and metalloproteinase with thrombospondin motifs (ADAMTs).^[^ ^4^ ^]^ However, in OA, the balance between the anabolism and catabolism of normal chondrocytes is disrupted, leading to overproduction of reactive oxygen species (ROS), inflammatory factors (IL‐1β, TNF‐β, IL‐6, and IL‐8), and catabolic factors (MMP1/3/13).^[^ ^5^ ^]^ Besides, during the latent initiation of OA, inflammatory chondrocytes are more susceptible to apoptosis.^[^ ^6^ ^]^ Therefore, strategies aimed at eliminating excess ROS and chondrocyte apoptosis, inhibiting inflammatory processes, and suppressing catabolic factor release are pivotal for OA treatment.

Recent therapeutic approaches for OA focus on preventing or delaying the destruction of articular cartilage and promoting regeneration by maintaining a balance between chondrocyte synthesis and metabolism. Ginsenosides, a class of natural compounds including triterpene saponins and steroid glycosides, have been identified as the primary active components in pharmacological investigations of ginseng.^[^ ^7^ ^]^ Approximately 40 ginsenosides have been discovered, with varying biological activities due to structural variations. Ginsenoside Rb1 (Gin) (Figure S1C, Supporting Information), the most common type, has been shown to have multiple biological properties such as anti‐inflammatory, anti‐apoptosis, and neuroprotective effects.^[^ ^8^ ^]^ Studies have demonstrated the promise of Rb1 in inhibiting ROS production and apoptosis in vascular endothelial cells,^[^ ^9^ ^]^ cardiomyocytes,^[^ ^10^ ^]^ and chondrocytes^[^ ^11^ ^]^ in vitro. In vivo, ginsenoside Rb1 has also been combined with scaffolds to attenuate inflammation and promote cartilage regeneration in OA rats.^[^ ^12^ ^]^

Despite these promising effects, a major challenge for most anti‐OA drugs (inflammatory inhibitors, growth factors, and anti‐matrix degradation molecules) is maintaining sustained and effective drug concentrations around chondrocytes to achieve the desired therapeutic effect.^[^ ^13^ ^]^ Although direct intra‐articular injection can increase local drug bioavailability and reduce adverse systemic side effects,^[^ ^14^ ^]^ drugs administered in this manner are rapidly cleared from the joint cavity through capillaries and lymphatic drainage.^[^ ^15^ ^]^ Furthermore, the small amount of drug remaining in the joint is subsequently excluded from the chondrocytes by dense and avascular cartilage extracellular matrix (ECM). The cartilage ECM, formed by chondrocytes, is composed of a dense network of cartilage collagen fibrils, aggrecan proteoglycans, and various extracellular macromolecules,^[^ ^16^ ^]^ which constitutes a formidable steric barrier despite its role in protecting chondrocytes from external stimuli. It also prevents the cartilage penetration of therapeutic compounds from synovial fluid and their subsequent absorption by chondrocytes, thereby limiting the bioavailability of drugs and greatly affecting the long‐term efficacy of OA.^[^ ^17^ ^]^ Therefore, there is a pressing need for a drug delivery system that can efficiently permeate into cartilage and bind to it before being cleared, converting the barrier of dense and avascular cartilage into a valuable drug reservoir for sustained intra‐tissue release.

A variety of nanoparticles, including liposomes, viral vectors, polymers, and inorganic nanomaterials, have been employed for drug delivery in OA treatment.^[^ ^18^ ^]^ However, challenges related to material compatibility, degradation, and cytotoxicity, as well as the host's immunological responses, limit their clinical applicability.^[^ ^19^ ^]^ DNA, known as a reservoir of genetic information for proteins and RNA synthesis, can self‐assemble into structurally diverse nanoparticles due to its unique properties, such as sequence programmability and molecular recognition.^[^ ^20^ ^]^ Since DNA nanostructures were first proposed in the 1980s, a considerable number of DNA nanostructures have been designed.^[^ ^21^ ^]^ Among these 3D nanoparticles, tetrahedral framework nucleic acids (tFNAs) are the simplest nanocarriers. They have garnered great attention and have been recognized as ideal nanocarriers for peptides, nucleic acids, small molecules, and monomers due to their good integrity and dispersibility, high programmability, strong cell internalization, and tissue penetration ability.^[^ ^22^ ^]^ Therefore, many studies have explored using tFNA to load a variety of small molecules and nucleic acid drugs for disease treatment. For example, some have reported the utilization of tFNA‐loaded curcumin, quercetin, and miRNAs for the treatment of osteoporosis, sepsis, and OA.^[^ ^19^, ^23^ ^]^ However, our previous studies have revealed that tFNA was mainly distributed on the cartilage surface and rarely penetrated the cartilage tissue in normal or OA knees after Cy5‐labeled tFNA injection in vivo (Figure 2E,I) thus impeding the application of tFNA in the treatment of OA diseases.

To overcome these limitations, we designed a novel tFNA‐based nanocarrier by combining the peptide WYRGRL (WL). It has been shown that this peptide can specifically bind type II collagen in cartilage and increase the cartilage targeting efficiency of peptide‐functionalized nanoplatforms in vivo by ≈72 times.^[^ ^24^ ^]^ Through click chemistry, followed by screening, a tFNA nanodrug delivery system (tFNA‐2WL) was then generated, capable of perfect cartilage penetration and excellent chondrocyte uptake (**Figure** **2**). Subsequently, the delivery system was loaded with Gin to finally synthesize cartilage‐targeted nucleic acid nanotherapeutic drugs (tFNA‐2WL&Gin) for targeted OA treatment (**Figure** **3**). Our results showed that tFNA‐2WL&Gin could effectively reverse the impaired chondrogenic ability of OA chondrocytes and inhibit cartilage degradation by simultaneously reducing apoptosis and remodeling the ROS microenvironment in vitro (**Figures** **4** and **5**). Besides, the nanomedicine exhibited an excellent performance in protecting the ECM in the joint capsule and cartilage, attenuating the hyperalgesia and stiffness, reducing the formation of osteophyte and normalizing subchondral bone of knees, and inhibiting inflammation and hypertrophy of synovial in the OA rats (**Figures** **6** and **7**). This study is expected to promote the delivery efficiency and efficacy of disease‐modifying biologic medicines for OA treatment and provide promising prospects in this field.

**Figure 1:**
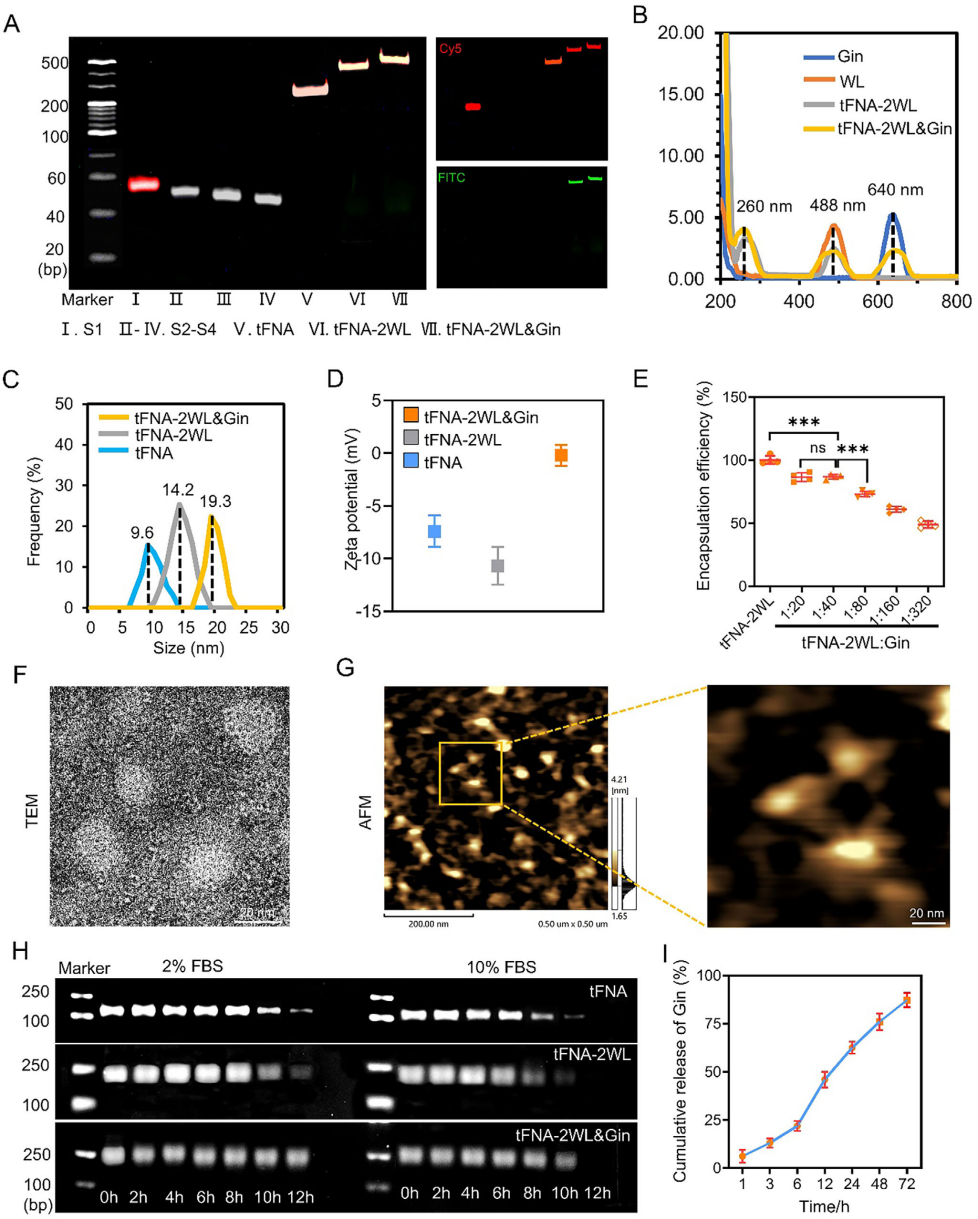
The synthesis and characterization of tFNA‐2WL and tFNA‐2WL&Gin. A) Characterization of the successful synthesis of tFNA‐2WL and tFNA‐2WL&Gin by PAGE. The results showed that the molecular weights of tFNA‐2WL and tFNA‐2WL&Gin were significantly larger than that of tFNA and the fluorescence of Cy5 in tFNA and Gin had a good colocalization with WL‐FITC (lane V‐VII) [S1 and Gin were labeled with Cy5 (red) and WL were labeled FITC (green)]. B) Spectra of Cy5‐Gin, WL(WL‐FITC), tFNA‐2W[tFNA‐2(WL‐FITC)] and tFNA‐2WL&Gin[tFNA‐2(WL‐FITC)&Cy5‐Gin] between 200 and 800 nm. C and D) Molecular diameters and zeta potential of tFNA, tFNA‐2WL, and tFNA‐2WL&Gin detected by DLS. E) Encapsulation efficiency of Gin loading on tFNA‐2WL. *n = 3*. F) The unique spatial structure of tFNA‐2WL&Gin was characterized by TEM. Scale bar, 20 nm. G) AFM images of tFNA‐2WL&Gin. Scale bar, 200 and 20 nm. H) Comparison of the stability of tFNA, tFNA‐2WL, and tFNA‐2WL&Gin in 2% and 10% FBS at different times by AGE. I) The release of Gin from tFNA‐2WL&Gin. The data are presented as the mean ± SD. Statistical differences were determined by one‐way ANOVA with Tukey's multiple comparisons test for (E).

**Figure 2:**
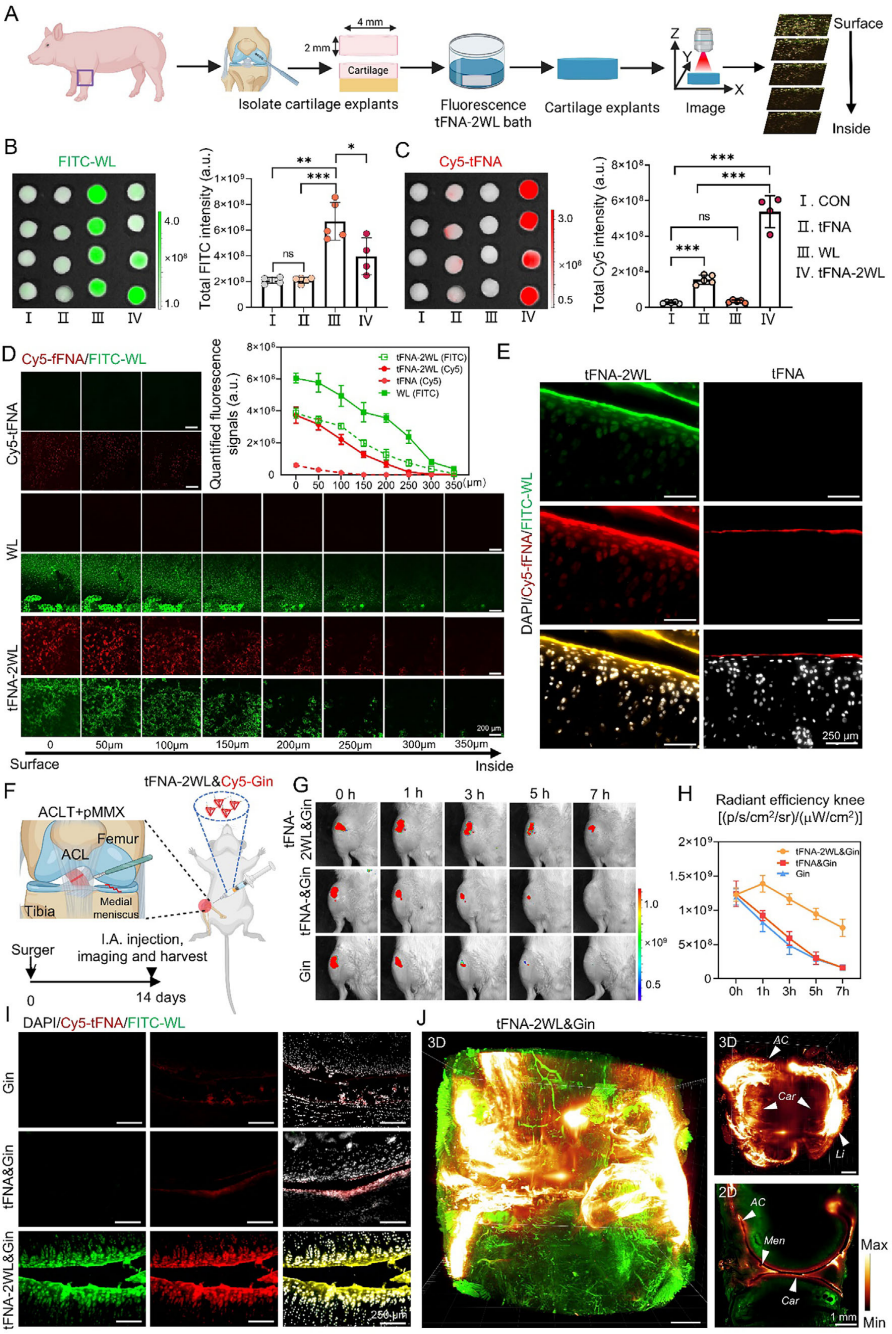
tFNA‐2WL and tFNA‐2WL&Gin can penetrate the normal and OA cartilage matrix deeply, in vitro and in vivo. A) Schematic illustration of porcine cartilage explant harvesting and detection of the penetration and distribution of fluorescent nanoparticles in cartilage via an IVIS and a confocal fluorescence microscope imaging. B and C) Fluorescence images and quantitative analysis of cartilage explants incubated with Control (PBS), tFNA (Cy5‐tFNA), WL(WL‐FITC), and tFNA‐2WL[Cy5‐tFNA‐2(WL‐FITC)] for 12 h using IVIS in FITC and Cy5 fluorescence channels, respectively. *n = 4*, *p < 0.05, **p < 0.01, ***p < 0.001. D) Horizontal fluorescence scanning images and quantitative analysis of these cartilage samples from (B) and (C) at different depths using a confocal microscope. The black arrow indicates the direction of nanoparticle penetration. *n = 4*, Scale bars, 200µm. E) Representative confocal microscopy images of cryosections harvested from normal rats 2 h after intra‐articular injection of tFNA (Cy5‐tFNA) and tFNA‐2WL [Cy5‐tFNA‐2(WL‐FITC)], respectively. *n = 3*. F) Schematic illustration of OA rats with surgery on the right hind knee (top) and timeline of intra‐articular injection of different nanomedicines. G and H) Representative IVIS images and quantitative analysis of OA rat knee joints over 7 h after injection of Gin (Cy5‐Gin), tFNA&Gin (tFNA&Cy5‐Gin), and tFNA‐2WL&Gin [tFNA‐2(WL‐FITC)&Cy5‐Gin] in vivo. *n = 3*. I) Representative confocal microscopy images of OA rat knee cryosections from (G). Scale bars, 250µm. J) Light‐sheet fluorescence microscopy three‐dimensional (3D) reconstruction images of the rat's OA joint with intra‐articular injection of tFNA‐2WL&Cy5‐Gin, after tissue clarity, and 3D fluorescence imaging by light‐sheet microscopy, showed that Cy5‐Gin fluorescence signals retained in cartilage, meniscus, ligament, and articular capsule (shown by white arrows). Note: yellow, Cy5‐Gin; green, tissue autofluorescence. *AC*: articular capsule, *Lig*: ligaments, *Men*: meniscus, *Car*: cartilage. Scale bars, 1 mm. All data are presented as the mean ± SD. Statistical differences were determined by one‐way ANOVA with Tukey's multiple comparisons test for (B), and (C).

**Figure 3:**
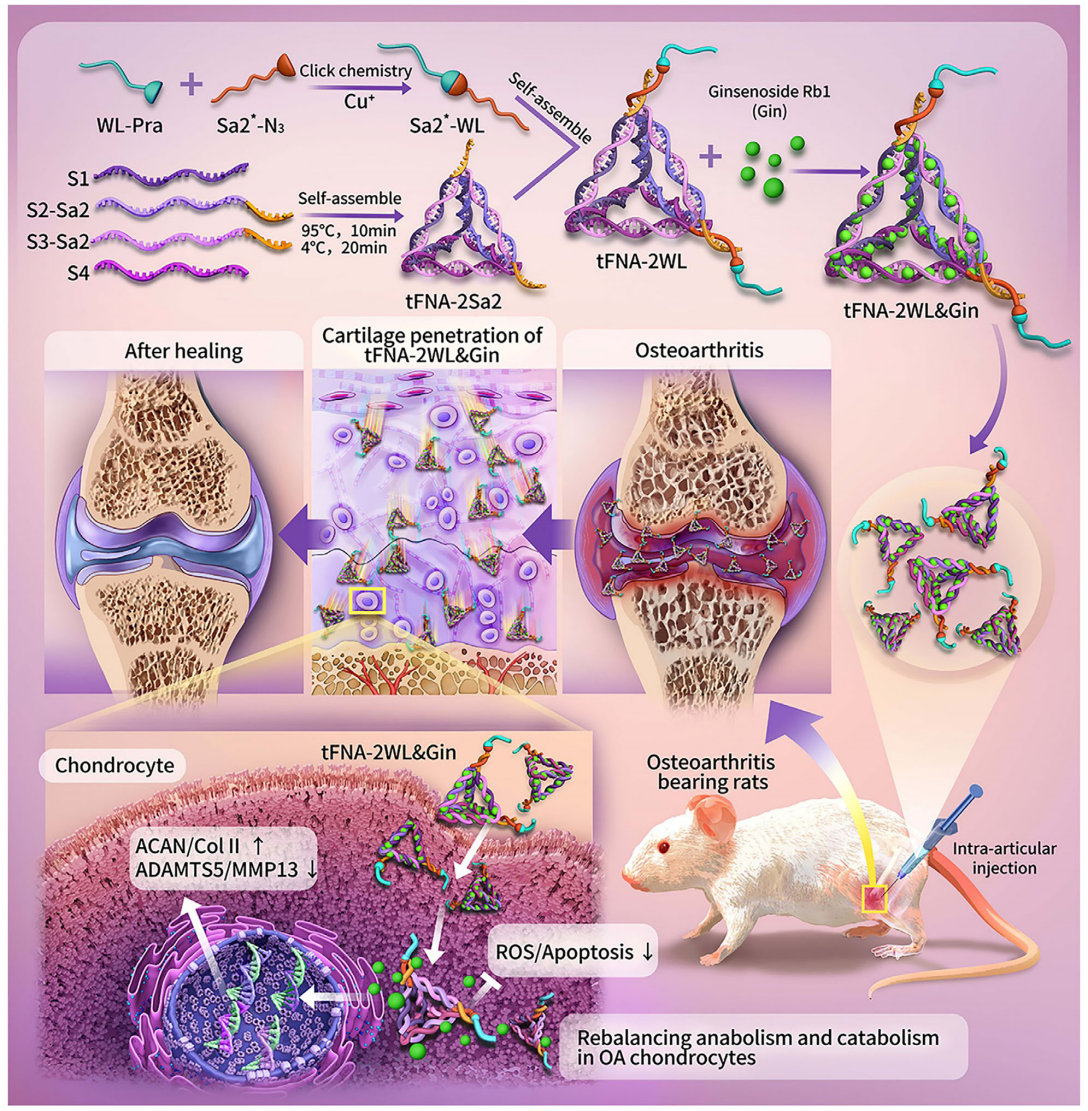
Schematic illustration. We designed and fabricated tFNA‐2WL and tFNA‐2WL&Gin via self‐assembly of bases and click chemistry. tFNA‐2WL&Gin was administrated into the knee joints of OA rats by intra‐articular injection. The tFNA‐2WL&Gin penetrated deeply into the cartilage matrix, was effectively uptake by chondrocytes, and sustainably released Gin to protect cartilage from osteoarthritic damage by rebalancing anabolism and catabolism in OA chondrocytes.

**Figure 4:**
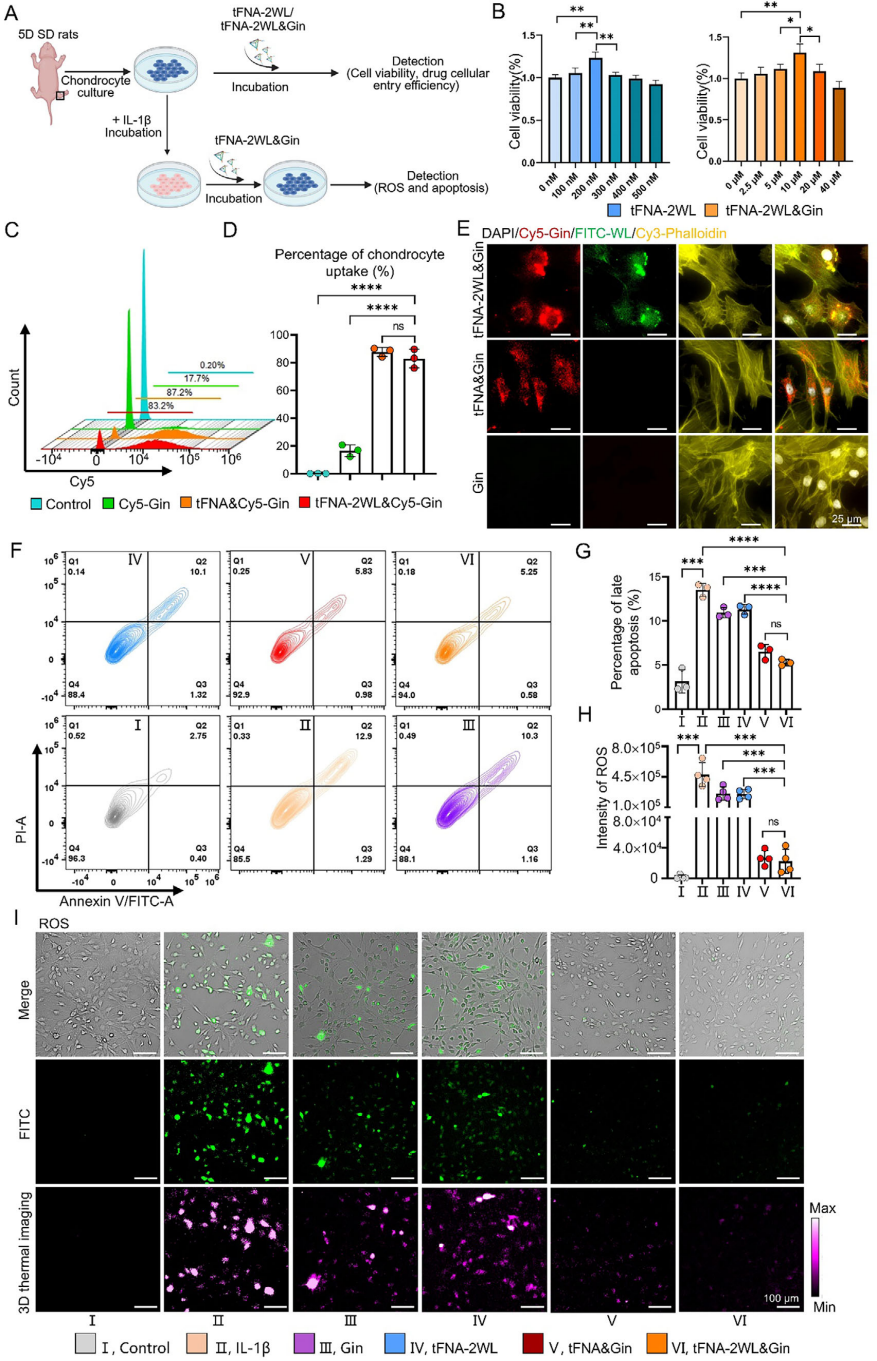
tFNA‐2WL&Gin can be efficiently uptake by chondrocytes, effectively reduce ROS, and significantly inhibit apoptosis of chondrocytes in vitro. A) Schematic illustration of extraction of chondrocytes, construction of OA chondrocyte model, characterization of chondrocyte uptake, and detection of the ability of nanomedicines to intervene in ROS and apoptosis of OA chondrocytes. B) Cytotoxicity of nanomedicine systems of tFNA‐2WL and tFNA‐2WL&Gin, characterized by CCK8 kit after co‐incubation of chondrocytes with different concentrations of tFNA‐2WL (0, 100, 200, 300, 400, 500 nm) and tFNA‐2WL&Gin (Gin: 0, 2.5, 5, 10, 20, 40 µm) for 12 h. C and D) Evaluation and quantitative analysis of uptake efficiency of Cy5‐Gin, tFNA&Gin (tFNA&Cy5‐Gin), and tFNA‐2WL&Gin (tFNA‐2WL&Cy5‐Gin) measured by flow cytometry. *n = 3*. E) Representative confocal fluorescence images of chondrocytes co‐cultured with nanomedicines of Cy5‐Gin, tFNA&Gin (tFNA&Cy5‐Gin) and tFNA‐2WL&Gin [tFNA‐2(WL‐FITC)&Cy5‐Gin] for 6 h. Scale bars, 250 µm. F and G) Representative fluorescence and quantitative analysis of the apoptosis in OA chondrocytes treated with IL‐1β, IL‐1β + Gin, IL‐1β + tFNA‐2WL, IL‐1β + tFNA&Gin, and IL‐1β + tFNA‐2WL&Gin was measured by flow cytometry. H and I) Quantitative analysis and fluorescence images of ROS in OA chondrocytes of the above groups (H) were detected by fluorescence imaging. Scale bars, 100µm. All data are presented as the mean ± SD. Statistical differences were determined by one‐way ANOVA with Tukey's multiple comparisons test for (B), (D), (G), and (H). *p < 0.05, **p < 0.01, ***p < 0.001, ****p < 0.0001.

**Figure 5:**
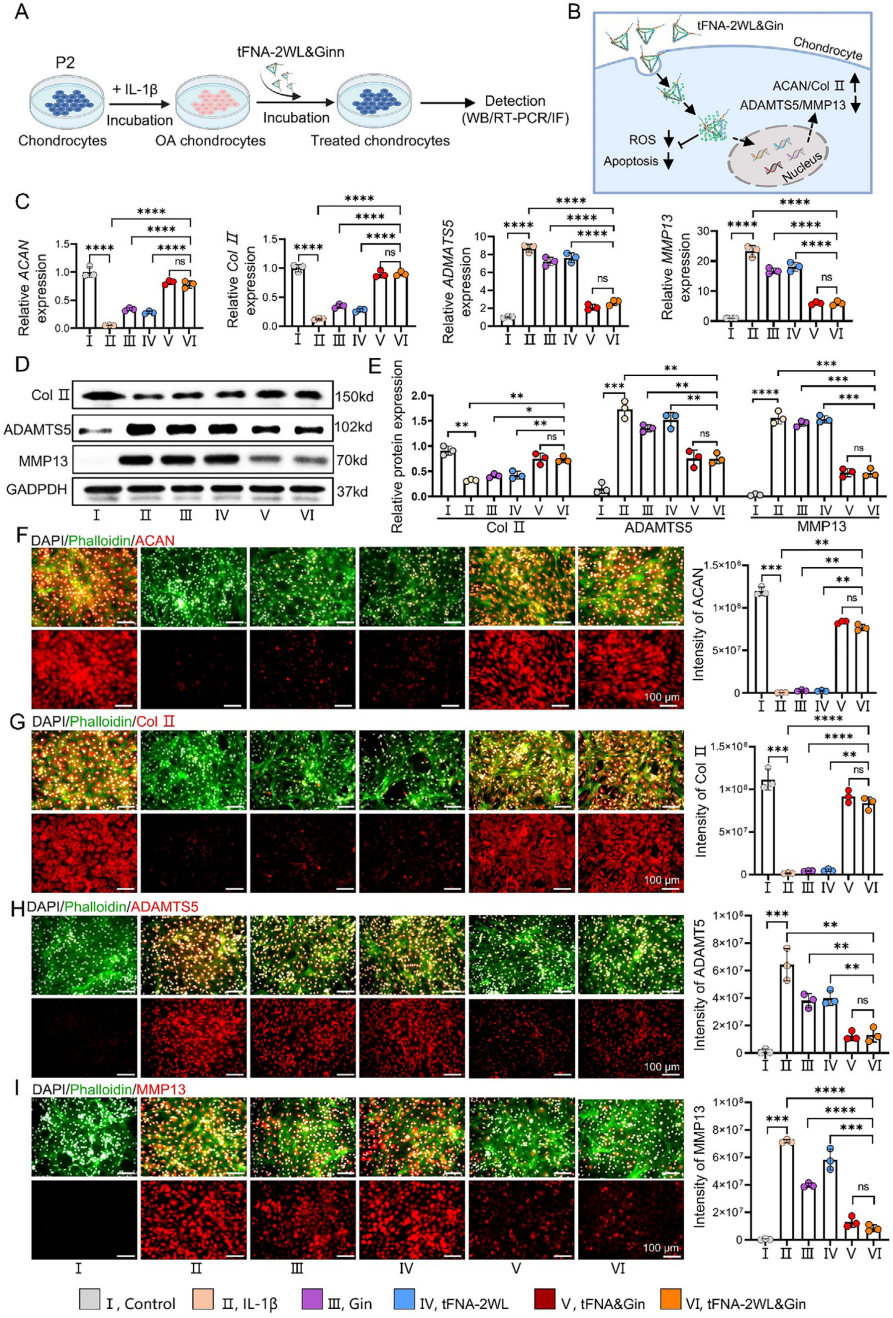
tFNA‐2W&Gin can effectively reverse the impaired chondrogenesis and reduce the cartilage degradation of OA chondrocytes. A) Schematic illustration of detection of chondrogenic and cartilage degradation abilities of OA chondrocytes after treatment with different nanomedicines. B) Schematic diagram of key processes getting involved in promoting impaired chondrogenesis and reducing the catabolism of OA chondrocytes of tFNA‐2W&Gin. C) RT‐PCR analysis of the gene expression of ACAN, Col II, ADAMTS5, and MMP13 of the chondrocytes in the above six groups. D and E) Western blotting analysis of the protein expression of Col II, ADAMTS5, and MMP13 of the chondrocytes in each group. F–I) Immunofluorescence images and quantitative analysis of ACAN, Col II, ADAMTS5, and MMP13 expression in chondrocytes after multiple drug treatments. *n = 3*, Scale bars: 100 µm. All data are presented as the mean ± SD. Statistical differences were determined by two‐way ANOVA for (E) and by one‐way ANOVA with Tukey's multiple comparisons test for (C), and (F–I). *p < 0.05, **p < 0.01, ***p < 0.001, ****p < 0.0001.

**Figure 6:**
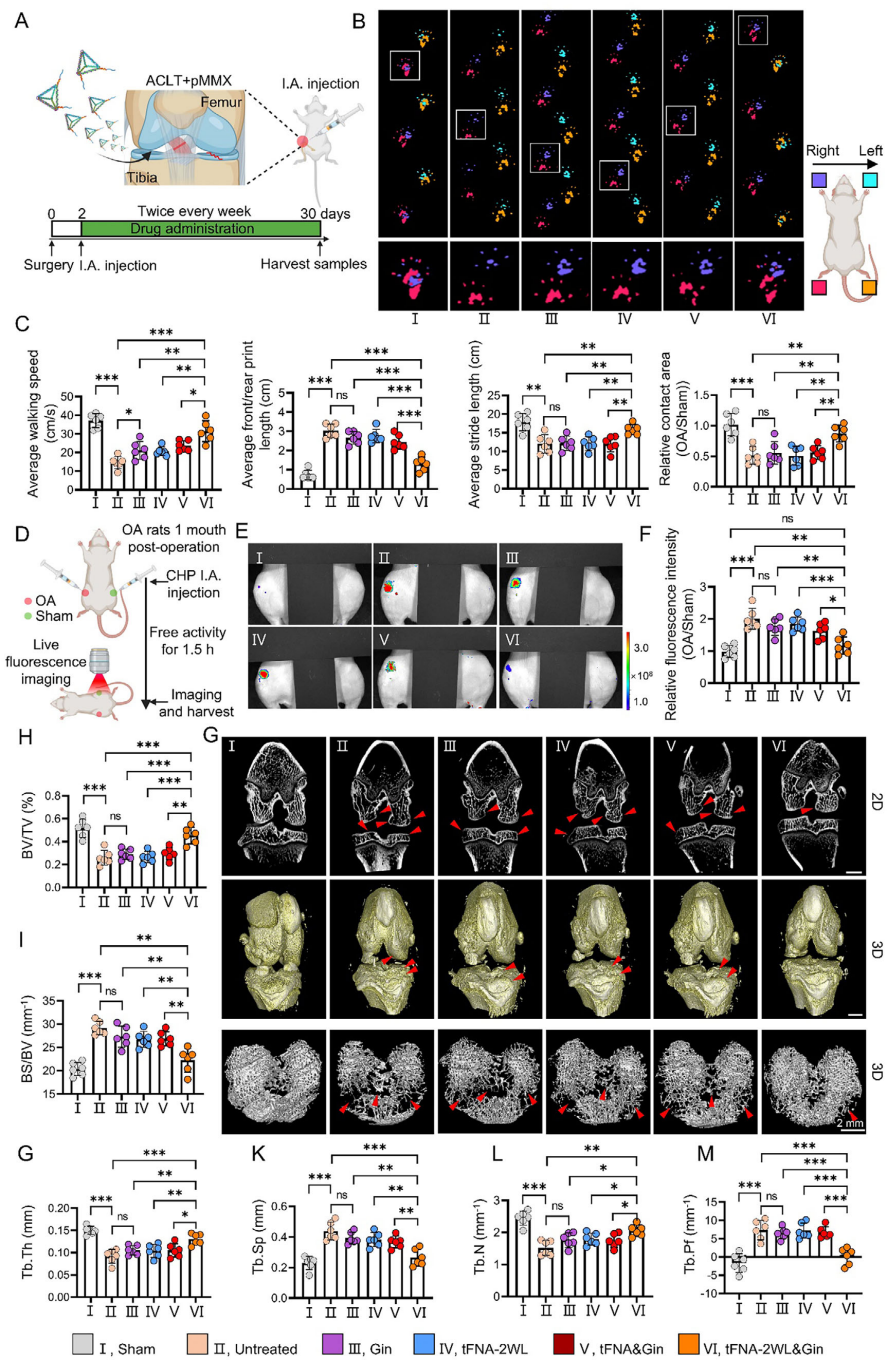
tFNA‐2W&Gin can attenuate the development of OA via relieving pain and gait, alleviating collagen tissue damage, and reducing osteophyte formation and subchondral bone resorption in OA rats. A) Schematic of the timeline and protocol of multiple treatments for OA rats induced by the ACLT + pMMx surgery. B) Representative photographs of the rats’ gaits after different treatments for 4 weeks. Left front foot, sky blue; Right front foot, purple; Left hind foot, yellow; Right hind foot, red. C) Quantitative analysis of average walking speed, average front/rear print length, average stride length, and relative contact area of the right hind limb of the rats after different treatments for 4 weeks. *n = 6*. D) Schematic of detection of joint collagen tissue damage for all rats after conditional treatment for 4 weeks by the intra‐articular injection of Cy5‐CHP in vivo. E and F) Representative IVIS images and quantitative analysis of rat knee joints with different treatments for 4 weeks after over 1.5 h by injection of Cy5‐CHP. *n = 6*. G) Representative 2D micro‐CT images (first row), 3D reconstruction images of knee joints (second row), and trabeculae of the tibial plateau (third row) showing the occurrence of osteophytes, cartilage and bone defects and bone resorption (shown by red arrows). *n = 6*, Scale bar, 2 mm. H–M) Quantitative analysis results of the parameters of subchondral bone microarchitecture (BV/TV, BS/BV, Tb.Th, Tb.Sp, Tb.N, and Tb.Pf) in the tibial epiphysis. All data are presented as the mean ± SD. Statistical differences were determined by one‐way ANOVA with Tukey's multiple comparisons test for (C), and (H–M). *p < 0.05, **p < 0.01, ***p < 0.001.

**Figure 7:**
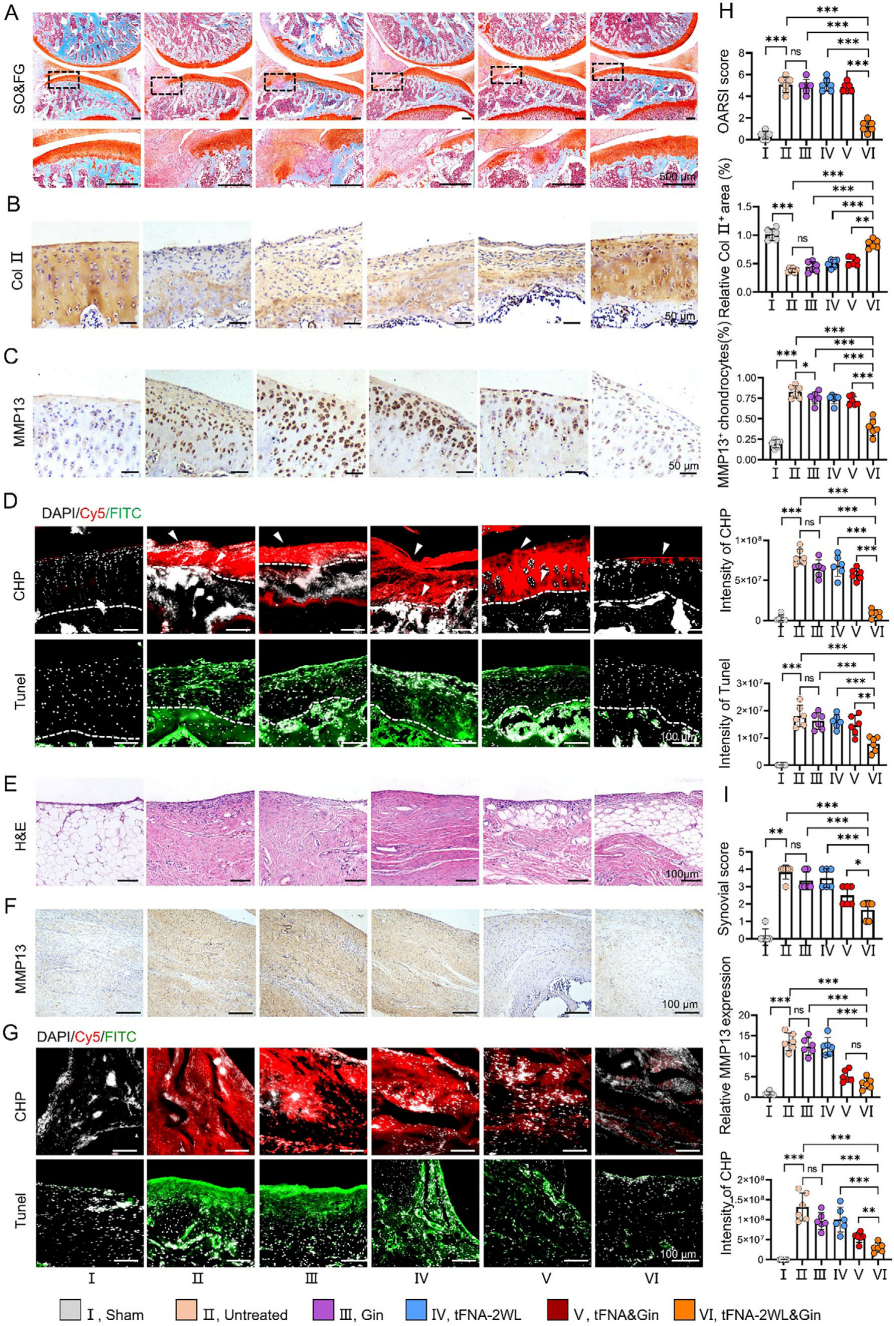
tFNA‐2W&Gin not only can significantly reduce cartilage degradation, and promote cartilage regeneration, but also alleviate synovial inflammation and Collagen degradation in OA rats. A–C) Representative images of SO&FG, Col II, and MMP13 staining of cartilage from the rats after 4 weeks of different treatments. Scale bars, A) 500 µm B,C) and 50 µm. D) Representative fluorescence images of CHP and Tunel staining of cartilage from the rats with conditional treatments. Scale bars, 100 µm. E and F) Representative images of H&E and MMP13 staining of the articular capsule tissue after 4 weeks of treatments. Scale bar, 100 µm. G) Representative fluorescence images of CHP and Tunel staining of articular capsule tissue from the rats after 4 weeks of treatments. Scale bars, 100µm. H) Quantitative analysis of cartilage degeneration was evaluated by OARSI score, Col II^+^ area, the rate of MMP13^+^ chondrocytes and fluorescence intensity of CHP, and quantitative of the intensity of Tunel fluorescence staining to explore the apoptosis in chondrocytes of each group. *n = 6*. I) Quantitative analysis of the inflammation, the expression of MMP13, collagen destruction, and apoptosis in synovium. *n = 6*. All data are presented as the mean ± SD, and statistical differences were determined by one‐way ANOVA with Tukey's multiple comparisons test for (H) and (I). *p < 0.05, **p < 0.01, ***p < 0.001.

## 2 Results and Discussion

### 2.1 Synthesis and Characterization of the tFNA‐nWL and tFNA‐2WL&Gin

As shown in Figure 3, the synthesis of tFNA‐nWL (*n* = 1–4) and tFNA‐2WL&Gin was divided into four steps. First, tFNA‐nSa2 (n = 1–4) was self‐assembled with four ssDNAs (Figure S3A and Table S1, Supporting Information) featuring specific sequences. Second, the Pra‐WL/Pra‐WL‐FITC (Figure 3) was conjugated with Sa*‐N3 via click chemistry to synthesize Sa2*‐WL/Sa2*‐WL‐FITC. In the subsequent step, tFNA‐nWL was synthesized via base complementary pairing of tFNA‐2Sa and Sa2*‐WL(Figure S3A, Supporting Information). Lastly, tFNA‐2WL&Gin was formed by mixing tFNA‐2WL and Gin (Figure S1C, Supporting Information) at a ratio of 1:40 and stirring the mixture at room temperature for 3 h. The successful formation of tFNA‐nWL/tFNA‐2WL&Gin was verified by fluorescence spectrum detection and polyacrylamide gel electrophoresis (PAGE) (Figure 3A; Figure S3A, Supporting Information). According to PAGE analysis, the position of tFNA‐nWL moved upward as the value of *n* increased, and the position of tFNA‐2WL&Gin further rose compared to tFNA‐2WL. Additionally, colocalization of Cy5‐S1 and FITC‐WL, as well as the presence of both Cy5‐Gin and FITC‐WL‐specific fluorescent signals in tFNA‐2WL&Gin, was observed (Figure 1A; Figure S3A, Supporting Information). The fluorescence spectrum detection revealed absorption peaks of tFNA (260 nm) and FITC‐WL (488 nm) in tFNA‐2WL and the absorption of tFNA, FITC‐WL, and Cy5‐Gin (640 nm) in tFNA‐2WL&Gin (Figure 1B). These results confirmed the successful synthesis of tFNA‐nWL and tFNA‐2WL&Gin.

To further characterize the physicochemical properties of those nanoparticles, dynamic light scattering (DLS) was used, finding that the diameters of tFNA, tFNA‐2WL, and tFNA‐2WL&Gin were 9.6, 14.2, and 19.3 nm, respectively (Figure 1C). For tFNAs, both WL modification and Gin loading increased their volume. Additionally, zeta potential measurements showed values of −7.4 ±1.36 mV for tFNA, −10.68 ± 1.62 mV for tFNA‐2WL, and −0.2 ± 0.9 mV for tFNA‐2WLs&Gin (Figure 1D). tFNA can bind to small molecule drugs through hydrogen bonding, charge interactions, and chimeric interactions with nucleic acid grooves.^[^ ^18^, ^25^ ^]^ Therefore, based on the structure of ginsenoside Rb1, we hypothesize that there are two main ways for it to bind to tFNA: first, the hydrogen bonds between the hydroxyl group in ginsenoside Rb1 and the guanine and cytosine residues of DNA; and second, it can also be bound with tFNA through charge interaction because of their own positive charge properties. Furthermore, atomic force microscopy (AFM) and transmission electron microscopy (TEM) images revealed that tFNA‐2WL and tFNA‐2WL&Gin retained a tetrahedral structure with sizes ranging from 10 to 20  nm (Figure 1F,G; Figure S2A–C, Supporting Information). To optimize the Gin loading in the tFNA‐2WL carrier, we performed encapsulation rate assay experiments. We found that the drug loading rate remained above 80% when the tFNA‐2WL to Gin ratio was below 1:40, but as the ratio increased beyond 1:40, the loading rate decreased. Therefore, 1:40 was the optimal ratio for subsequent experiments (Figure 1E). The stability of tFNA‐2WL and tFNA‐2WL&Gin in serum was assessed using agarose gel electrophoresis (AGE). tFNA‐2WL&Gin exhibited greater stability than that of free tFNA and tFNA‐2WL as it remained intact in serum for ≈12 h, with no significant difference in stability at 2% and 10% serum concentrations (Figure 1H). Moreover, there was also no significant difference in the serum stability between tFNA and tFNA‐2WL in both 2% and 10% fetal bovine serum (FBS). The above results indicated that Gin loaded on tFNA‐2WL did not destabilize the nanostructure and that WL modification did not compromise the overall stability of the delivery system. Besides, the release kinetics of Gin from tFNA‐2WL&Gin were evaluated, demonstrating a slow and sustained release over 72 h. After 24 h, 60% of the Gin was released (Figure 1I), which was conducive to prolonged therapeutic action.

### 2.2 The Excellent Cartilage Penetration of the tFNA‐2WL and tFNA‐2WL&Gin In Vitro and In Vivo

tFNA has attracted much attention due to its strong drug loading, excellent tissue infiltration, and endocytosis abilities, but our previous studies found that the cartilage penetration of tFNA is insufficient. Therefore, we combined peptide WL, which can specifically bind to cartilage, with tFNA to compensate for the inherent drawbacks of tFNA, and thus tFNA‐nWLs (Figure S3A, Supporting Information) were born. Considering that nanomedicines must be able to penetrate the cartilage ECM before being taken up by chondrocytes, we then studied the relationship between cartilage ECM and nanoparticles. We first investigated the cartilage penetration of tFNA‐nWL (*n* = 1–4) by co‐incubation with cartilage explants in vitro (Figure 2A). After 10 h of incubation with tFNA‐nWL [Cy5‐tFNA‐n(FITC‐WL)], the samples were washed with PBS and scanned by fluorescence imaging and confocal microscopy. The images showed that the tFNA‐2WL‐incubated cartilage with a strong FITC fluorescence signal showed the strongest Cy5 fluorescence signals compared with the other groups, and the quantitative analysis results also yielded consistent results (Figure 2B,C; Figure S4A,B, Supporting Information). Confocal horizontally and vertically scanned images of cartilage explants revealed that tFNA‐2WL demonstrated the best penetration into cartilage compared to the other four groups (Figure S4C, Supporting Information). The penetration depth of tFNA‐2WL was close to 350 nm from the surface of cartilage, which was comparable to the WL, and was almost 7 times that of tFNA (Figure 2D). The right knee joints of six normal rats were injected with equal amounts of tFNA and tFNA‐2WL via intra‐articular injection and subjected to dynamic fluorescence imaging for 4 h to investigate the metabolism and retention of tFNA‐2WL in vivo. tFNA‐2WL showed a significantly longer retention time than that of tFNA (Figure S5A–D, Supporting Information). By performing cryo‐sections on these above‐knee joints to explore the specific retention of the two nanocarriers in the cartilage, we found that tFNA‐2WL could penetrate the middle layers of the cartilage and enter chondrocytes, while tFNA only aggregated on the cartilage surface (Figure 2E). In conclusion, the cartilage penetration of tFNA could be significantly enhanced by combining it with WL, and tFNA‐2WL had a perfect cartilage penetrating capability both in vitro and in vivo.

To further explore the penetration of tFNA‐2WL&Gin in cartilage, OA rats at 2 weeks postoperatively were selected and injected intra‐articularly with Cy5‐Gin, tFNA&Cy5‐Gin, and tFNA‐2WL&Cy5‐Gin, followed by fluorescence imaging, harvesting, and histopathological examination (Figure 2F). To ensure the equal quantity of nanoparticles utilized in each group, all nanoparticles were injected with an equal amount of Gin (10 µm, 50 µL) and tFNA (250 nm, 50 µL). The results showed that tFNA‐2WL&Gin had the longest retention time in OA joints during the 7 h of test, followed by tFNA&Gin, while Gin had the fastest metabolism among the three groups (Figure 2G). Quantitative analysis of the fluorescence signal of Cy5‐Gin in OA knee joints showed that tFNA&Gin and Gin demonstrated a faster clearance rate after drug injection and completely disappeared after 5 h post‐injection. In contrast, the fluorescence intensity of tFNA‐2WL&Gin increased 1 h after drug injection and showed a slower attenuation (Figure 2H). In addition, when Cy5‐Gin had been completely cleared in normal or OA joints of the Gin group at the 7th h after drug injection, there was still significant Cy5‐Gin residue in both joints in the tFNA‐2WL&Gin group, with significantly stronger fluorescence signals in the OA joints compared to the Sham side (Figure S6A, Supporting Information). Quantitative analysis showed that the intensity of fluorescence signals in bilateral knee joints in the tFNA‐2WL&Gin group was two times more than that of the other two groups, and the fluorescence in the OA knee joints was the highest (Figure S6A,B, Supporting Information). To clarify the cartilage penetration of Gin in each OA group, cryo‐sections were performed. We discovered that compared to the other two groups in which no significant fluorescence was revealed in cartilage, the tFNA‐2WL&Gin group showed extremely strong fluorescence signals of Cy5‐Gin and FITC‐WL in cartilage and chondrocytes, with good colocalization (Figure 2I). The results demonstrated that tFNA‐2WL&Gin could fully penetrate OA cartilage and was effectively taken up by chondrocytes. The above studies have only shown the retention of tFNA‐2WL&Gin in articular cartilage. The joint, as a 3D space, contains more tissues except for the cartilage, and the specific spatial distribution of tFNA‐2WL&Gin in OA joints needs to be further explored. Tissue clearing and 3D fluorescence imaging can assist us in resolving the above problems, as this technique enables 3D visual imaging of opaque tissues and organs by making tissue transparent.^[^ ^26^ ^]^ By intra‐articular injection of the three drugs into the OA joints in vivo, followed by tissue clearing and 3D fluorescence imaging, we had a deeper understanding of the biodistribution of tFNA‐2WL&Gin in the OA joints. The data showed that tFNA‐2WL&Gin was not only abundantly retained in the cartilage, but also in the meniscus, the joint capsule, and the articular ligaments (Figure 2J). In contrast, tFNA&Gin was only marginally enriched in the joint capsule. (Figure S6C and Videos S1–S3, Supporting Information). These results confirmed the superiority of tFNA‐2WL in prolonging the retention of nanocarriers within the joint and suggest that tFNA‐2WL has the potential to be a cartilage‐targeted nanomedicine delivery system.

### 2.3 tFNA‐2WL&Gin are Effectively Taken up by Chondrocytes, Significantly Reduce ROS, and Inhibit Apoptosis of OA Chondrocytes

Ginseng is a plant substance that is widely utilized in Asian countries as both a nutritional and a therapeutic ingredient. Highly effective plant products such as steroid glycosides and triterpene saponins, known as ginsenosides, are major active ingredients in medicinal studies of ginseng.^[^ ^7^ ^]^ Ginsenoside‐Rb1 (Gin), as one important representative of ginseng, not only has good biological safety,^[^ ^27^ ^]^ but also owns a variety of biological properties such as anti‐inflammatory, anti‐apoptotic, neuroprotective, anti‐arthritic, and antitumor, facilitating its wide application in the treatment of various diseases.^[^ ^8^, ^28^ ^]^ Some studies have reported that ginsenoside‐Rb1 could inhibit apoptosis and reduce inflammation in human or rabbit articular chondrocytes.^[^ ^29^ ^]^ However, because of the dense and avascular nature of articular cartilage, poor cartilage penetration and inefficient cellular uptake of drugs are important factors that compromise the efficacy of OA therapy, and ginsenoside‐Rb1 is no exception to this rule. Previous studies demonstrated that tFNA could effectively load small molecules and achieve efficient intracellular delivery.^[^ ^13^, ^17^, ^23^, ^30^ ^]^ Besides, tFNA‐2WL, which was synthesized in the previous stage with good cartilage permeability, was endowed with WL and the same efficient cellular entry characteristics as tFNA. Therefore, we envisioned loading Gin on tFNA‐2WL to promote the intracellular delivery of the drugs and thus enhance their therapeutic efficacy on OA chondrocytes. First, according to the studies,^[^ ^19^, ^31^ ^]^ we isolated chondrocytes from the knee joints of suckling SD rats (Figure 4A; Figure S7, Supporting Information) and incubated them with different concentrations of Gin, tFNA, tFAN‐2WL, and tFAN‐2WL&Gin for 24h. Cytotoxicity of the drugs was then detected by CCK8 and the results showed that the optimal concentration of both tFNA and tFAN‐2WL was 200 nm, while that of Gin, tFNA&Gin, and tFNA‐2WL&Gin was 10 µm (Gin) (Figure 4B; Figure S8A, Supporting Information). Second, we used flow cytometry and microscopy to detect the uptake of Gin, tFNA‐2WL, and tFNA‐2WL&Gin by chondrocytes. Based on the results of the flow cytometry, the percentage of fluorescence‐detectable cells of tFNA group and tFNA‐2WL group both reached up to 86% (Figure S8B, Supporting Information). In addition, the uptake efficiency of tFNA&Gin and tFNA‐2WL&Gin (both above 80%) was significantly higher than that of Gin alone (17.7%), a more than five‐fold improvement (Figure 4C,D). Similar results were also obtained from the fluorescence images with characteristic fluorescent signals of tFNA‐2WL and tFNA‐2WL&Gin broadly distributed in the cytoplasm (Figure 4E; Figure S8C, Supporting Information). These data showed that both tFNA‐2WL and tFNA‐2WL&Gin had perfect cellular entry properties, thus laying the foundation for the subsequent efficient treatment of OA.

In OA chondrocytes, increased oxidative stress can inhibit autophagy and induce apoptosis.^[^ ^32^ ^]^ In apoptotic chondrocytes, mitochondrial dysfunction can further promote the production of ROS, which in turn creates a vicious cycle that exacerbates the development of OA. In addition, increasing evidence shows that there is a strong correlation between apoptosis and cartilage damage.^[^ ^33^ ^]^ Therefore, cell apoptosis becomes an important potential target for OA therapy. To mimic the microenvironment of OA in vitro, IL‐1β (20 ng mL^−1^), as a major catabolic cytokine in OA, was co‐cultured with chondrocytes to induce an OA chondrocyte model. Flow cytometry analysis was used to evaluate the anti‐apoptosis effects of Gin, tFNAs, tFNAs&Gin, and tFNA‐2WL&Gin on OA chondrocytes. The data showed that compared with the weak anti‐apoptotic effects of Gin (10.9 ± 0.59%) and tFNA (11.3 ± 0.55%) on OA chondrocytes (13.5 ± 0.74%), tFNA&Gin (6.5 ± 0.83%) and tFNA‐2WL&Gin (5.3 ± 0.35%) could significantly reverse the IL‐1β‐induced chondrocyte apoptosis, and there were not statistical differences in the anti‐apoptotic effects between these two groups (Figure 4F,G). Excessive production of ROS can induce oxidative stress to damage the DNA in chondrocytes and, in turn, stimulate cell apoptosis.^[^ ^34^ ^]^ In addition, high levels of ROS also inhibit the regeneration of articular cartilage and tissue.^[^ ^35^ ^]^ Therefore, it is particularly important to prevent cartilage damage in OA by scavenging ROS. In the study, the production of ROS in chondrocytes of different groups was measured by fluorescent imaging. The data showed that chondrocytes in the IL‐1β, Gin, and tFNA‐2WL groups exhibited significant ROS fluorescence signals, with the strongest fluorescence signals in the IL‐1β group. Conversely, the fluorescence signals in the tFNA&Gin and tFNA‐2WL&Gin groups were both significantly reduced to the same level as that in the control group (Figure 4I). Quantitative analysis found that the fluorescent intensity in the tFNA&Gin and tFNA‐2WL&Gin groups was ≈20‐fold lower compared to IL‐1β, and ≈15‐fold lower compared to the Gin group (Figure 4H). The above experimental results fully demonstrated that the anti‐apoptotic effect and ROS inhibition of Gin on OA chondrocytes could be significantly enhanced by loading them onto tFNA and tFNA‐2WL.

### 2.4 tFNA‐2WL&Gin Promote Chondrogenesis of OA Chondrocytes and Inhibit Cartilage Degradation

The development of OA is a process of imbalance between chondrocyte anabolism and catabolism, which is accompanied by the degradation of the ECM of articular cartilage.^[^ ^19^ ^]^ MMP13 and ADAMTS5 play pivotal roles in maintaining the stability of the cartilage extracellular matrix (ECM) in OA. Studies have found that the expression of both MMP13 and ADAMTS5 is increased in the cartilage of OA patients or animals, and the downregulation of the above markers has been shown to prevent cartilage degeneration in animal OA models.^[^ ^5^, ^36^ ^]^ In vitro, we proved the feasibility of increasing the uptake efficiency of Gin by loading them onto tFNA and tFNA‐2WL, meanwhile significantly inhibiting cell apoptosis and ROS production in OA chondrocytes, but the influence of tFNA&Gin and tFNA‐2WL&Gin on the chondrogenesis of OA cartilage and cartilage degradation should be further explored (Figure 5A). First, qRT‐PCR results showed that the mRNA expressions of collagen II (Col II) and aggrecan (ACAN) were significantly reduced, while an increase in the expression of OA‐related catabolic mediators (i.e., MMP13 and ADAMTS5) was noted in IL‐1β‐treated chondrocytes in the IL‐1β, Gin, and tFNA‐2WL treated groups. However, the above phenomenon caused by IL‐1β was effectively reversed by tFNA&Gin and tFNA‐2WL&Gin (Figure 5C). Similar results were further verified by Western Blotting analysis. After tFNA&Gin and tFNA‐2WL&Gin treatment, the expression of Col II in OA chondrocytes, which was previously reduced by IL‐1β, was significantly increased, while the expression of MMP13 and ADAMTS 5, which were enhanced before, was significantly inhibited (Figure 5D,E). Immunofluorescence staining showed that IL‐1β inhibited the production of ACAN and Col II and promoted the production of MMP13 and ADAMTS5, as evidenced by the decreased or increased red fluorescence intensity in IL‐1β‐treated chondrocytes (Figure 5F–I). Compared with the weak fluorescence in the Gin and tFNA‐2WL groups, the tFNA&Gin and tFNA‐2WL&Gin groups presented extremely strong fluorescence signals, indicating that tFNA&Gin and tFNA‐2WL&Gin possessed a stronger capability to promote the expression of ACAN and Col II in IL‐1β‐stimulated chondrocytes compared to Gin and tFNA‐2WL (Figure 5F,G). As shown in Figure 5H,I, tFNA&Gin and tFNA‐2WL&Gin significantly reduced the high fluorescence intensity of MMP13 and ADAMTS5 induced by IL‐1 β, whereas the inhibitory effect of Gin and tFNA‐2WL were minimal. The results indicated that tFNA&Gin and tFNA‐2WL&Gin with increasing cellular uptake via loading Gin onto tFNA or tFNA‐2WL regained the balance between anabolic and catabolic metabolism of OA by inhibiting ROS production, resisting apoptosis, thus increasing the synthesis of anabolic factors (ACAN/Col II) and decreasing the secretion of catabolic factors (MMP13/ADAMTS 5) of OA chondrocytes (Figure 5B).

### 2.5 tFNA‐2WL&Gin Can Effectively Alleviate OA In Vivo

To test the in vivo therapeutic potential of tFNA‐2WL&Gin, we performed serial drug treatments on OA rats and comprehensively evaluated the therapeutic effects by gait analysis, IVIS fluorescence imaging, micro‐CT, and histopathological assay (Figure 6A). First, an OA rat model was constructed by performing anterior cruciate ligament transection (ACLT) and partial medial meniscectomy (pMMx) on the right hind limb.^[^ ^13^, ^17^, ^19^ ^]^ Two days after surgery, these rats were randomly divided into six groups, five of which underwent surgeries and received intra‐articular injections of PBS, Gin, tFNA‐2WL, tFNA&Gin, and tFNA‐2WL&Gin, respectively. The rats in the last group underwent sham surgery and were regarded as the control. After 4 weeks of treatment, we first investigated the parameters of footprint analysis, and found that compared to Gin, tFNA‐2WL, and tFNA&Gin, the Intra‐articular injection of tFNA‐2WL&Gin significantly increased the walking speed, stride length, and the right hind limb contact area, meanwhile reducing the front/rear print length in the ACLT+pMMX‐injured rats (Figure 6B,C; Figure S9B and Video S4, Supporting Information). Based on the above experimental results, we speculated that tFNA‐2WL&Gin could effectively attenuate hyperalgesia and stiffness of OA joints to alleviate dyskinesia in rats.

As the major structural component of the ECM in almost all mammalian tissues and organs, collagen plays an important role in supporting cell attachment, proliferation, migration, and differentiation.^[^ ^37^ ^]^ Col II is the predominant component of cartilage ECM, and its degradation is an important molecular event during OA. Therefore, the destruction and degradation of cartilage collagen also serve as an important indicator for the disease monitoring of OA.^[^ ^38^ ^]^ The collagen‐hybridizing peptide (CHP) can specifically bind to the denatured collagen strands by reforming a triple–helical structure.^[^ ^39^ ^]^ It has been proved that the single‐strand CHPs could specifically hybridize with unfolded collagen chains damaged or enzymatically cleaved but with negligible affinity to intact collagen.^[^ ^40^ ^]^ CHPs also enable direct screen degradation of collagen from isolated tissues of osteoarthritis, myocardial infarction, and pulmonary fibrosis.^[^ ^38c^ ^]^ Therefore, to verify the effect of the nanomedicines on inhibiting cartilage degradation in OA, CHP (10 µm, 100 µL) was injected into the joints of OA rats after 4 weeks of treatment and detected by the IVIS fluorescence imaging system (Figure 6D). The results showed that the fluorescence signal of the OA side knee of rats in group tFNA‐2WL&Gin was significantly weaker than that of the other four groups (untreated, Gin, tFNA‐2WL, and tFNA&Gin), although it was slightly increased compared with that of the left knee both in vivo and in vitro. The enhanced fluorescence signal in the OA‐side knee joint was not statistically significant compared with the sham group by quantitative analysis (Figure 6E,F; Figure S10A, B, Supporting Information), which indicated that tFNA‐2WL&Gin also efficiently alleviated the collagen damage in the knee joints of OA rats.

Bone response plays an important role in the occurrence and development of osteoarthritis.^[^ ^41^ ^]^ Therefore, we used micro‐computed tomography (Micro‐CT) to assess the therapeutic effect of nanomedicines on periarticular bone, namely, osteophyte development and tibial subchondral bone remodeling. In the representative 2D micro‐CT images, the characteristic imaging manifestations of osteophytes were protrusions of normal bone contours, decreased bone density, and a reduced number of trabeculae. The results showed that the number of osteophytes formed in the knee joints from the groups of untreated, Gin, tFNA‐2WL, and tFNA&Gin was significantly greater than that of tFNA‐2WL&Gin (indicated by red arrows in Figure 6G and white arrows in Figure S11, Supporting Information). In addition, 3D reconstructions of the knee and the trabeculae in the tibial plateau showed rough surface morphology, decreased trabecular volume, and widened intertrabecular spaces in the groups of untreated, Gin, tFNA‐2WL, and tFNA&Gin, compared with that of the tFNA‐2WL&Gin group (Figure 6G, shown by the red arrow). We further analyzed the tibia subchondral bone microarchitecture parameters, including the ratio of bone volume to tissue volume (BV/TV), the bone surface‐to‐volume ratio (BS/BV), the trabecular thickness (Tb. Th), the trabecular spacing (Tb. Sp), trabecular number (Tb. N) and trabecular pattern factor (Tb. Pf), to evaluate the subchondral bone remodeling. Compared with the sham group, the microstructure parameters of BV/TV, Tb. N, and Tb. Th was obviously increased, while the parameters of BS/BV, Tb. Sp, and Tb. Pf was decreased in the untreated group, which was barely changed by the injection of Gin, tFNA‐2WL, or tFNA&Gin. However, tFNA‐2WL&Gin treatment effectively decreased BV/TV, Tb. N, and Tb. Th and increased BS/BV, Tb. Sp, and Tb. Pf in the ACLT+pMMX induced OA rats (Figure 6H–M). Together, these data suggested that tFNA‐2WL&Gin could efficiently hinder osteophyte formation and protect the subchondral bone of knee joints in OA rats.

Moreover, we performed histological analysis using Safranin O–Fast Green (SO&FG), H&E, Masson, immunohistochemistry, and immunofluorescence staining to assess cartilage structural changes. Those rats receiving treatments of PBS, Gin, tFNA‐2WL, or tFNA&Gin presented a similar pattern of cartilage damage with proteoglycan loss, fibrillation, and even severe cartilage erosion (Figure 7A; Figure S12, Supporting Information, shown by black arrow), leading to Osteoarthritis Research Society International (OARSI) scores of 5.08 ± 0.74, 4.91 ± 0.66, 5.08 ± 0.58, and 4.83 ± 0.41, respectively (Figure 7A,H). The differences among the four groups in OARSI scores were not significant (*p > 0.05*). In contrast, the group treatment with tFNA‐2WL&Gin remarkably improved the cartilage structure and morphology (1.25 ± 0.52, OARSI score), resulting in a nearly intact cartilage surface with only minor proteoglycan loss (Figure 7A,H). The results of immunohistochemistry staining showed that the decreased expression of Col II and the increased expression of MMP13 under OA were not meaningfully ameliorated by the treatment of Gin, tFNA‐2WL, or tFNA&Gin. On the contrary, tFNA‐2WL&Gin effectively inhibited the reduction of Col II and the overexpression of MMP13 (Figure 7B,C; Figure S13, Supporting Information). Subsequently, we performed frozen sections on the knee cartilage of OA rats labeled with CHP in vivo (Figure 6D) and found that the entire layer of damaged cartilage in the untreated, Gin, tFNA‐2WL, and tFNA&Gin groups, were enriched with abnormally rich CHP fluorescence, while in the tFNA‐2WL&Gin group, only a relatively weak fluorescence signal was detected on the surface of cartilage (as shown in Figure 7D, white arrow), further indicating that tFNA‐2WL&Gin could effectively alleviate cartilage damage in OA. In addition, the results of articular chondrocyte apoptosis measured by Tunel staining were consistent with CHP staining. In the untreated, Gin, tFNA‐2WL, and tFNA&Gin groups, the number of Tunel‐positive chondrocytes increased significantly and showed a strong FITC fluorescence signal. In contrast, the number of Tunel‐positive chondrocytes in the tFNA‐2WL&Gin group was significantly reduced, with a weak fluorescence signal correspondingly (Figure 7D), which demonstrated that tFNA‐2WL&Gin possessed a more pronounced inhibit apoptosis of OA chondrocytes. These results indicate that the tFNA‐2WL&Gin can effectively protect articular cartilage from damage and alleviate the development of OA.

We further evaluated the therapeutic effect of tFNA‐2WL&Gin on synovitis by analyzing the inflammation scoring following the histopathology initiative guidelines of OARSI^[^ ^42^ ^]^ and synovial hyperplasia based on H&E and Masson staining. These joints treated with PBS, Gin, or tFNA‐2WL demonstrated a large number of inflammatory cell infiltration, the remarkable hyperplasia of synovial tissue, and thickened synovial lining cell layers (Figure 7E; Figure S14A, Supporting Information), with a more than ≈3.5‐fold higher inflammation score compared with the sham group (*p < 0.001*) (Figure 7I). In contrast, tFNA&Gin and tFNA‐2WL&Gin treated joints exhibited 2.5‐ and 1.7‐fold increases, respectively, indicating that tFNA&Gin and tFNA‐2WL&Gin possessed good anti‐inflammatory effects and that tFNA‐2WL&Gin had better performance. Subsequently, the immunohistochemistry and the immunofluorescence staining of MMP13, CHP, and Tunel were performed to evaluate the synovial inflammation and the collagen tissue damage in the joint capsule (Figure 7F,G). The MMP13 protein expression. The fluorescence of CHP and tunel staining in the joint capsule was significantly higher in the OA group than that in the sham group. The above overexpression could not be suppressed by the Gin, tFNA‐2WL, but was effectively downregulated by tFNA&Gin and tFNA‐2WL&Gin, with tFNA‐2WL&Gin showing a more pronounced inhibitory effect (Figure 7F,G,I; Figure S14B, Supporting Information). In summary, tFNA&Gin and tFNA‐2WL&Gin could both alleviate inflammation and prohibit hyperplasia, tissue damage, and cell apoptosis in OA joint capsule synovial. We speculated that this might be related to the tissue penetration and cell entry properties of tFNA&Gin and tFNA‐2WL&Gin, and the longer drug retention time of tFNA‐2WL&Gin in the OA joints eventually led to its better therapeutic efficacy.

Lastly, we evaluated the potential systemic side effects of the nanoparticles in rats by conducting histopathological analyses of the major organs from all rats. Compared with the rats from the sham group, we did not detect any tissue damage or abnormalities in all of the organs that were harvested from the rats with intra‐articular injections of Gin, tFNA‐2WL, tFNA&Gin, and tFNA‐2WL&Gin (Figure S15, Supporting Information). These results indicated that the pharmacotherapy for OA via intra‐articular injection of tFNA‐2WL&Gin is reliable, with good biosafety and minimal systemic toxicity.

## 3 Conclusion

In this study, we first designed and synthesized a tetrahedral framework nucleic acid nanomedicine delivery system (tFNA‐2WL) with good cartilage penetration and cell entry properties and then loaded Gin on this system to treat OA rats effectively by rebalancing the anabolic and catabolic metabolism of OA chondrocytes. Due to the perfect affinity of WL to cartilage and the unique framework structure of tFNA, the tFNA‐2WL had better performance in cartilage targeting, cartilage penetration, and uptake of chondrocytes. In our study, compared to tFNA, tFNA‐2WL had a nearly seven‐fold increase in the penetration depth into the cartilage, with the same excellent cell entry properties as tFNA. We also loaded Gin onto the tFNA‐2WL system and synthesized tFNA‐2WL&Gin. Compared to Gin and tFNA&Gin, the tFNA‐2WL&Gin could efficiently deliver Gin into cartilage and enter OA chondrocytes with high transfection efficiency and enhanced therapeutic effect both in vitro and in vivo. The in vitro experiments suggested that the tFNA‐2WL&Gin system exhibited anti‐inflammatory and anti‐apoptotic capabilities, inhibition of IL‐1β‐induced cartilage degradation, and promotion of cartilage regeneration. Besides, the tFNA‐2WL&Gin presented a favorable performance in protecting the ECM in the joint capsule and cartilage against damage, attenuating synovial tissue hyperalgesia and stiffness, reducing osteophyte formation, and protecting the subchondral bone structure of OA knees. Despite the powerful efficacy of the tFNA‐2WL&Gin, one of the main challenges for the nano platform is long‐term biosafety, which requires further systematic study. In addition, according to the structure design, tFNA‐2WL might also be combined with siRNA or micro‐RNA to be utilized for OA gene therapy, one possibility that also needs to be further explored. Furthermore, the mechanism of how tFNA‐2WL&Gin reversed the impaired chondrogenic ability of OA chondrocytes and inhibited cartilage degradation could be further investigated. In summary, the designed tFNA‐2WL&Gin originated from the imperative clinical demand, which will provide a promising paradigm for the future development and application of targeted drug therapy of OA.

## 4 Experimental Section

### Materials

The designed single‐stranded oligonucleotides (Cy5‐S1, S1, S2, S3, S4, S1‐Sa2, S2‐Sa2, S3‐Sa2, S4‐Sa2, and Sa2*‐N3) (shown in Table S1, Supporting Information) and all primer sequences in our study (shown in Table S2, Supporting Information) were synthesized and purchased from Sangon Biotechnology (Shanghai, China). The WL and WL‐FITC modified with lysine‐terminated propargylglycine (Pra) (Pra‐WL/Pra‐WL‐FITC) (Figure S1A,B, Supporting Information) and Cy5‐CHP (shown in Table S1, Supporting Information) were designed by ourselves and synthesized by Chinese Peptide Biochemical Co. Ltd.

### Synthesis of tFNA‐nWL and tFNA‐2WL&Gin

First, according to the method reported previously,^[^ ^22^, ^25^ ^]^ equal amounts (100 µm, 1 µL) of four single‐stranded oligonucleotides with specific sequence (Figure 3A; Figure S3A and Table S1, Supporting Information) were added to microtubes containing 96 µL TM buffer composed of equimolar amounts of Tris‐HCl and MgCl_2_ to synthesize Cy5‐tFNA‐nSa2, tFNA‐nSa2 (*n* = 1–4), Cy5‐tFNA‐2Sa2, and tFNA‐2Sa2 via self‐assemble following the procedure (95 °C, 10 min; 4 °C, 20 min). Second, Pra‐WL and Pra‐WL‐FITC were combined with Sa2*‐N_3_ to synthesize Sa2*‐WL and Sa2*‐WL‐FITC by click chemistry. 119.44 µL of DMSO, 40 µL of 10 mm CuSO_4_, 14.16 µL of 28.3 mm Tris (benzyltriazolylmethy) amine (TBTA), and 40 µL of 10 mm ascorbic acid were mixed and set aside for 5 min, Subsequently, added 40 µL, 200 µm Sa2*‐N_3_, and 26.4 µL, 600 µm Pra‐WL/Pra‐WL‐FITC to the system and incubated overnight at room temperature. Finally, the initial product of WL‐Sa2*/FITC‐WL‐Sa2* was purified. On the second day, a specific volume of NaAc and anhydrous ethanol were added to the original WL‐Sa2*/FITC‐WL‐Sa2* solution at a volume ratio of WL‐Sa2*/FITC‐WL‐Sa2*: NaAc (3M): anhydrous ethanol of 10:1:25. After mixed, the solution was stored at −20 °C for 30 min, then centrifuged at 14 rpm for 15 min at 4 °C, removed the supernatant and added 1.5 mL of 70% ethanol, centrifugated at 14 rpm for 10 min to obtain the precipitate, and then stood it at room temperature for 20 min. After the ethanol was volatilized completely, 100 µm of WL‐Sa2*/FITC‐WL‐Sa2* solution was obtained by adding 80 µL of deionized water. Finally, Cy5‐tFNA‐nSa2/tFNA‐nSa2 and Sa2*‐WL/Sa2*‐WL‐FITC were combined through the base complementary pairing of Sa2 and Sa2* to obtain Cy5‐tFNA‐n(WL‐FITC), Cy5‐tFNA‐2WL, Cy5‐tFNA‐2(WL‐FITC) or tFNA‐2(WL‐FITC). A certain amount of Cy5‐tFNA‐nSa2/tFNA‐nSa2 was mixed with 1.25n times the amount of Sa2*‐WL/Sa2*‐WL‐FITC and incubated at room temperature for 6 h to obtain the nanomaterials. Cy5‐Gin or Gin (Figure S1C, Supporting Information) was mixed with tFNA or tFNA‐2WL in the ratio of 1:40 and shaken at room temperature for 3 h to obtain tFNA‐2WL&Gin, tFNA‐2WL&Cy5‐Gin, tFNA‐2WL&Gin, and tFNA‐2WL&Cy5‐Gin.

### Characterization of tFNA‐nWL and tFNA‐2WL&Gin

To verify whether tFNA‐nWL and tFNA‐2WL&Gin were successfully synthesized, the examines of PAGE, HPCE, DLS, AFM, and TEM were performed to detect their structures. First, the approximate relative molecular weights of these nanocomplexes were determined using 8% native PAGE. The sizes and zeta potentials of tFNA‐2WL&Gin and tFNA‐2WL were measured by dynamic light scattering (DLS, Nano ZS, Malvern, England). AFM (Libra200, Zeiss, Oberkochen, Germany) and TEM (Cypher VRS, Oxford Instruments, UK) were used to characterize the shapes and sizes of tFNA‐2WL&Gin and tFNA‐2WL. The ultra‐microspectrophotometer (UV5 Nano, Mettler Toledo, Switzerland) was utilized to detect the absorbance spectra of tFNA, Cy5‐Gin, tFNA‐2(WL‐FITC), and tFNA‐2(WL‐FITC)&Cy5‐Gin.

### Assay of Encapsulation Efficiency, Drug Release, and Stability of the Nanocomplexes

Equal volumes of tFNA‐2WL (1 µm) and Cy5‐Gin with different concentrations (20, 40, 80, 160, 320 µm) were mixed and vibrated at room temperature for 3 h. The excess Cy5‐Gin was then removed by molecular weight filter (30 kDa, Millipore, USA) and measured by OD value at a wavelength of 647 nm. For the release experiments, 1 × PBS (0.01 mol L^−1^, pH 7.4) was used as the buffer medium. 3 mL of PBS solution containing the equivalent of Cy5‐Gin and tFNA‐2WL&Cy5‐Gin (40 µm) was added to a dialysis membrane (30 kDa, Solarbio, Beijing), which was sealed and placed in a beaker. Then, 30 mL of PBS (0.01 mol L^−1^) was added to the outside. The entire system was placed at 37 °C, and the amount of Cy5‐Gin released in the external solution was determined by the OD values at specific time points (1, 3, 6, 12, 24, 48, 72 h). To detect the stability of tFNA‐2WL and tFNA‐2WL&Gin in serum, a certain amount of tFNA‐2WL and tFNA‐2WL&Gin (tFNA‐2WL: 250 nm) was added to a complete culture medium containing 2% and 10% fetal bovine serum, respectively, and co‐cultured at 37 °C for 0, 2, 4, 6, 8, and 12 h, and then agarose gel electrophoresis (AGE) was performed. Lastly, the degradation of the nanoparticles was detected by an ultraviolet exposure instrument (Bio‐Rad, Hercules, USA).

### Porcine Cartilage Explant Harvest and Culture

All operations in this study were performed after review and approval by the Institutional Review Board of Sichuan University and strictly followed the requirements of relevant ethical principles. Articular cartilage explants were harvested from the trochlear groove of porcine forelimbs and cultured according to the methods of the studies.^[^ ^13^, ^30^, ^43^ ^]^ Next, 250 µm, FITC‐WL, Cy5‐tFNA, or Cy5‐tFNA‐n(WL‐FITC) (*n* = 1–4) were incubated with the cartilage explants for 12 h. After washing with PBS, the samples were then fixed with 4% PFA for 24 h. The final step was to image and quantify these samples using an IVIS (Spectrum, PerkinElmer) and a confocal fluorescence microscope (Olympus, Tokyo, Japan) (Figure 2A).

### Near‐Infrared In Vivo Fluorescence Imaging

All near‐infrared fluorescence imaging in this study was performed using an IVIS Spectrum imager (PerkinElmer Lumina III). For the retention assay of tFNA‐2WL in the knee joint, 100 µL of 1 µm Cy5‐tFNA and Cy5‐tFNA‐2(WL‐FITC) in sterile isotonic saline was injected into the right knee joint of SD rats (8 weeks old). The living image software (PerkinElmer) was used to serially detect and quantify radiant efficiency within each joint at 0, 1, 2, 3, and 4 h after injection. At 4 h after injection, the rats were euthanized, and the knee joints were harvested for IVIS imaging to assess the retention of tFNA‐2WL in normal joints ex vivo.

To examine knee joint retention and cartilage penetration of tFNA‐2WL&Gin, 12 rats with OA for 2 weeks (10 weeks old, right knee joint was OA side and left knee joint was Sham side) were selected and injected with Cy5‐Gin, tFNA&Cy5‐Gin, and tFNA‐2(WL‐FITC)&Cy5‐Gin into bilateral joints, respectively (*n* = 3 for each group, 10 µm, 100 µL, Cy5‐Gin for each knee joint). Subsequently, fluorescence imaging was performed on the bilateral knee joints of rats at 0, 1, 3, 5, and 7 h after injection to continuously observe the metabolism of tFNA‐2(WL‐FITC)&Cy5‐Gin in joints. At the seventh hour, the bilateral knee joints of rats in each group were obtained for ex vivo imaging, and then frozen sections of the OA knee joints were performed to observe the enrichment and penetration of tFNA‐2(WL‐FITC)&Cy5‐Gin in OA cartilage. The protocol of in vivo imaging and frozen sections was the same as those described above.

To investigate the damage of the knee joint tissues in each group of treated rats, Cy5‐CHP (10 µm, 100 µL) was injected into the bilateral knee joints of all rats. After the injection, the rats were allowed to move freely, and fluorescence imaging was performed 1.5 h later. Next, their knee joint specimens were collected, imaged, and conducted for histopathological analysis. The acquired images were analyzed using the living image software.

### Cryosections of the Knee Joint

To accurately observe the distribution of the nanoparticles and the detection probe (CHP) in the knee joint after near‐infrared fluorescence imaging in vivo, the samples need to be subjected to cryosectioning and fluorescence imaging. First, these collected knee joints were fixed in 4% PFA for 48 h and then decalcified with 20% EDTA (w/v) solution for 14 days until the tissue was completely decalcified. These samples were then sequentially dehydrated in 30% (w/v) sucrose solution and 30% (w/v) sucrose + 30% (w/v) optimal cutting temperature compound (OCT) mixed solution for 12 h, respectively. After that, the tissues were embedded in OCT and prepared into 15 µm frozen sections. Finally, these cryosections would be detected by fluorescence microscopy.

### Tissue Clarity and Light‐Sheet Fluorescence Microscopy Imaging

Exploring the specific retention of tFNA‐2WL&Gin in OA knee joints, we injected Cy5‐Gin, tFNA&Cy5‐Gin, and tFNA‐2WL&Cy5‐Gin into the right joint of OA 2 W rats as the previous experiment, harvested samples 7 h later, and performed tissue clearing and 3D fluorescence imaging on the knee joints of each group following the way reported in the study.^[^ ^44^ ^]^ At 7 h after injection, the rats were perfused transcardially by a heparinized PBS solution to remove blood. Next, the OA knee samples were collected and cleared according to the PEGASOS method.^[^ ^26^ ^]^ First, knee joint samples were fixed with 4% PFA at room temperature for 48 h and then decalcified with 20% EDTA for 2 weeks to complete the decalcification. Then, they were decolorized with 25% Quadrol (122262, Sigma) solution for 2 days. After that, they were degreased with 30%, 50%, and 70% tert‐butyl (TB) alcohol (360538, Sigma) for 3 days, respectively, and then dehydrated with TB‐PEG solution [75% TB + 22% PEGMEMA500 (447943, Sigma) + 3% Quadrol] for 2 days. Finally, the specimens were immersed in clearing medium‐BB‐PEG [75% benzyl benzoate (W213802, Sigma) + 22% PEGMEMA500 + 3% Quadrol] for clarity. Cleared rat joints were imaged by the Zeiss Lightsheet 7 microscope. The samples were imaged with 638 nm (Cy5‐Gin) and 488 nm (autofluorescence) lasers. The stitching of the images was done using the Arivis software. The 3D reconstruction images and videos were produced by using the Imaris software (Figure 2J; Figure S6C and Videos S1–S3, Supporting Information).

### Extraction of Chondrocytes and Construction of OA Chondrocytes

According to previous studies,^[^ ^45^ ^]^ chondrocytes were harvested from the articular cartilage of newborn male SD rat knee joints (5 to 6 days old) by shearing and enzymatic digestion with type II collagenase. Briefly, chondrocytes were collected from neonatal SD rat knee cartilage by shearing it into pieces and enzymatically digesting it with 0.2% type II collagenase for 5 h at 37 °C in an incubator, followed by centrifugation at 350 × g for 10 min. The collected chondrocytes were resuspended with a complete medium containing 89% DMEM, 10% FBS, and 1% antibiotics (penicillin–streptomycin), placed in T25 culture flasks, and incubated in a 37 °C, CO_2_ incubator. In this study, chondrocytes were used from passages 2. The OA chondrocyte model was established using IL‐1β (20 ng/mL) co‐incubated with chondrocytes for 12 h. Subsequently, chondrocytes were then supplemented with Cy5‐Gin, Gin, Cy5‐tFNAs‐2WL, tFNA&Gin, or tFNA‐2WL&Gin (Gin: 10 µm, tFNA/ tFNA‐2WL: 250 nm) for 12 h of incubation and then subjected to further detection.

### The Chondrocytes' Uptake of tFNA‐2WL and tFNA‐2WL&Gin

The P2 chondrocytes were seeded into 24‐well plates (1 × 10^5^ cells/mL) and cultured for 12 h and then were cocultured with Cy5‐tFNA and Cy5‐tFNA‐2(WL‐FITC) (250 nm), Cy5‐Gin, tFNA&Cy5‐Gin and tFNA‐2(WL‐FITC)&Cy5‐Gin (10 µm) for 6 h. For flow cytometry analysis, chondrocytes were first washed with PBS, digested with EDTA‐free trypsin, centrifuged and collected, then resuspended in cold PBS and analyzed using a flow cytometer (FC500, Beckman Co., USA). For fluorescence imaging, the chondrocytes of each group were washed with PBS to remove the culture medium, and fixed with 4% PFA for 20 min, followed by perforating using 0.5% Triton X‐100 for 10 min, and then stained with AF568‐labeled phalloidin (1:500; A12380, Thermo Fisher) and DAPI (1:1000; C1002, Beyotime). Finally, after washing three times with PBS, these chondrocytes were imaged by a confocal microscope (Leica TCS SPE, Germany) and analyzed by ImageJ.

### Cellular Apoptosis and ROS Detection

To explore the effect of tFNA‐2WL&Gin to reduce ROS and anti‐apoptosis of OA chondrocyte. According to the experiment, there were six groups, including control, IL‐1β, Gin, tFNA‐2WL, tFNA&Gin, and tFNA‐2WL&Gin. Among them, the control group was normal chondrocytes without IL‐1β treatment, and chondrocytes in the IL‐1β group were co‐cultured with IL‐1β (20 ng/mL) for 24 h. Those chondrocytes in groups of Gin, tFNA‐2WL, tFNA&Gin, and tFNA‐2WL&Gin were treated with IL‐1β for 12 h and then separately incubated with IL‐1β+Gin, IL‐1β+tFNA‐2WL, IL‐1β+tFNA&Gin, and IL‐1β+tFNA‐2WL&Gin (Gin: 10 µm, tFNA/tFNA‐2WL: 250 nm) for 12h. To detect the level of ROS of all chondrocytes, these cells were washed three times with PBS and incubated with DCFH‐DA solution (1:1000; S0033, Beyotime Biotechnology, China) for 20 min, and then imaged by confocal microscopy. Furthermore, the specific fluorescence intensity was analyzed by Image J and thermal images. Apoptosis levels in chondrocytes of all groups were measured via flow cytometry. Chondrocytes (4 × 10^5^ cells mL^−1^) were cultured in six‐well plates and co‐incubated with the different nanoparticles above following the protocol previously described,^[^ ^19^ ^]^ and then detected using flow cytometry after washing and digestion.

### Quantification of RT‐PCR

Total RNA from chondrocytes was purified with an RNeasy Mini Kit and reverse‐transcribed into complementary DNA using a cDNA synthesis kit (11752250, Thermo Fisher) according to the manufacturer's protocol. The RT‐PCR was performed to detect the target mRNA expression using the SYBR Premix Ex Tag Kit (RR390A, TaKaRa). The relative mRNA expression (ΔΔCt) of targeted genes was normalized by the expression of GAPDH. The primer sequences of targeted genes used in this study are shown in Table S2 (Supporting Information).

### Western Blotting Analysis

The targeted proteins, which were collected from chondrocytes of all groups with lysis buffer, were extracted through SDS–PAGE, transferred onto PVDF membranes, and blocked by 5% non‐fat dry milk. Then, they were incubated with anti‐GAPDH (1:500; 5174S, CST), anti‐Col II antibodies (1:1000; PA5‐99159, Thermo Fisher), anti‐MMP13 antibodies (1:500; 18165‐1‐AP, Thermo Fisher), and anti‐ADAMTS 5 antibodies (1:500; ER1903‐32, HUABIO) at 4 °C overnight, and then processed the incubation with the secondary antibody (1:2000; C31460100, Thermo Fisher) for 1 h. The enhanced chemiluminescence WB system (P0018S, Beyotime) was used to visualize the antibody reactivity.

### Immunofluorescence Staining of Chondrocytes

After treatment with different reagents (Gin, tFNA‐2WL, tFNA&Gin, and tFNA‐2WL&Gin), the chondrocytes were washed with PBS three times, followed by fixation with 4% paraformaldehyde for 20 min, perforated using 0.5% Triton X‐100 for 15 min, and blocked by 5% serum. Subsequently, the chondrocytes were incubated with the primary antibodies against Col II (1:300), ACAN (1:250; DF7561, Affinity Biosciences), MMP13 (1:300), and ADMATS5 (1:250) at 4 °C overnight. Next, chondrocytes were incubated with a Cy3‐Goat Anti‐Rabbit IgG secondary antibody (1:500; A10520, Thermo Fisher) for 1 h and stained again with FITC‐labeled phalloidin and DAPI. Lastly, the chondrocytes would be imaged using a confocal microscope.

### Surgical OA Model in Rats

To simulate severe human OA, OA rat models were established by performing anterior cruciate ligament transection (ACLT) and partial medial meniscectomy (pMMx) on the right hind limbs of 8‐week‐old male SD rats, according to previous studies^[^ ^13^, ^17^ ^]^ (Figure 6A). Briefly, an incision was created with a blade next to the patella on the medial side of the right hind limb to expose the joint, and then the joint capsule was opened by a longitudinal incision. After fully exposing the joint cavity using sutures pulling the patellar ligament to the lateral side, the ACL, medial meniscotibial ligaments, and the midpoint of the medial meniscus were transected, and then the tissue was repositioned and sutured. For animals of the sham group, only the joint capsule was opened to expose the meniscus and ACL, but no surgical operation was performed; the rest of the operations were the same as for the OA animals. All rats were divided into six groups according to the different treatments: I) no surgery with PBS treatment (sham), II) surgery with PBS treatment (untreated), III) surgery with Gin treatment (Gin), IV) surgery with tFNA‐2WL treatment (tFNA‐2WL), V) surgery with tFNA&Gin treatment (tFNA&Gin), and VI) surgery with tFNA‐2WL&Gin treatment (tFNA‐2WL&Gin). Two days after surgery, these nanomedicines were administered by intra‐articular injection at a frequency of twice a week. The dosage of injection for each knee joint was 50 µL of PBS, Gin (10 µm), tFNA‐2WL(250 nm), tFNA&Gin(Gin:10 µm), and tFNA‐2WL&Gin (Gin:10 µm). After treatment administration for 30 days, all rats were euthanized after relevant tests, and the knee joints and major internal organs (heart, liver, spleen, lung, and kidney) were harvested for subsequent testing and analysis.

### Footprint Analysis

The parameters of the footprint analysis were measured by the GAT‐100 Gait Analysis System (Taimeng Software Co., Ltd, Chengdu, China). The gait analysis system uses a unique footprint recognition image algorithm to collect animal footprints and automatically distinguish the left front foot (sky blue), right front foot (purple), left hind foot (yellow), and right hind foot (red) in different colors. The rats were tested and analyzed after treatment administration for 4 weeks according to the methods reported in the literature,^[^ ^46^ ^]^ and three consecutive trials were performed on every animal. The images of all experiments were processed and analyzed by GAT‐100 software to obtain these gait parameters of average walking time, average front/rear print length, stride length, and the relative contact area (maximum contact area of right hind limb/maximum contact area of left hind limb) (Figure S9A, Supporting Information).

### Micro‐Computed Tomography of Knee Joints

After fixation in 4% PFA for 2 days and before decalcification, rat knee joints were positioned inside the bore of a Micro‐CT scanner [VNC‐102, PINGSENG Healthcare (Kunshan) Co. Ltd. China] and scanned at a voltage of 90 kV X‐ray potential and 50 µA current. Image datasets were reconstructed and analyzed using the associated analyzing software Avatar [PINGSENG Healthcare (Kunshan) Co. Ltd. China] with the FDK algorithm. The reconstruction pixel and dimension were 0.023 × 0.023 × 0.027 mm and 2000 × 2000 × 1134, respectively. To evaluate the total volume of the osteophytes in each joint, the contours of osteophytes in each 2D frontal slice were manually outlined, followed by 3D reconstruction and total volume calculation. For subchondral bone remodeling analysis, the histomorphometric evaluation was performed in the 3D positioning data of the tibial subchondral bone, including the ratio of bone volume to tissue volume (BV/TV), the bone surface‐to‐volume ratio (BS/BV), trabecular number (Tb. N), trabecular separation (Tb. Sp), trabecular thickness (Tb. Th), and trabecular pattern factor (Tb. Pf).

### Histological, Immunohistochemistry, and Immunofluorescence Staining and Analysis

All knee joints harvested from rats in this study were fixed in 4% PFA for 4 days and decalcified with 20% EDTA for 14 days at 37 °C. First, imaging the in vivo bound Cy5‐CHP with a fluorescence microscope (Figure 6D,F), the fixed and decalcified knee joints were cryo‐sectioned longitudinally to 15 µm thick, stained with DAPI, and imaged. After the frozen section, the remaining knee joint tissue was washed with PBS to remove OCT, followed by embedding in paraffin to obtain 5 µm sections to evaluate the histopathological features of joints by Safranin O–Fast Green staining, H&E staining, Masson staining, immunohistochemistry staining, and immunofluorescence staining according to the manufacturer's instructions. To evaluate the degradation of cartilage, OARSI, bone erosion, and bone formation were applied and analyzed the images of Safranin O–Fast Green staining, H&E staining, and Masson staining. According to the study,^[^ ^13^ ^]^ inflammation scoring and synovial hyperplasia were measured to evaluate the degree of synovitis via the H&E staining and Masson staining. Immunohistochemistry and immunofluorescence staining were performed following the standard methods. The knee joint sections were first incubated with primary antibodies (anti‐Col II, 1: 300; anti‐MMP13, 1: 300) overnight, followed by the incubation of secondary antibodies, and the diaminobenzidine substrate was performed for imaging. Tunel staining was performed using an apoptosis detection kit (Takara) to detect the apoptosis of cells in each group in vivo. All sections were imaged by the confocal microscope (Leica TCS SPE, Germany) and analyzed by ImageJ. The evaluation of all joint histopathological features was performed by two independent investigators in a blinded manner.

### Statistical Analysis

All the experiments in this study were conducted independently, at least in triplicate, and experimental data were presented as mean ± SD. One‐ and two‐way analyses of variance (ANOVA) were performed to assess the significant differences for three or more groups. A two‐tailed Student's *t*‐test was used to compare the statistical significance of the two groups. The statistical significance was shown as *p < 0.05, **p < 0.01, ***p < 0.001, ****p< 0.001. All data were analyzed by GraphPad Prism v.8.

## Conflict of Interest

The authors declare no conflict of interest.

## Author Contributions

K.H., Q.M.L. and H.X.L. contributed equally to this work. K.H., S.R.S., J.G.X., and Y.F.L. designed the experiments. K.H., Q.M.L., H.X.L., Q.S., and T.R.T. synthesized and characterized the materials. K.H., Q.M.L., H.X.L., and C.M. conducted cell experiments in vitro. K.H., Q.S., C.M., and Y.P.W. performed the animal experiments in vivo. K.H., Q.M.L., H.X.L. and C.S. organized and analyzed the data. K.H., Q.M.L. and H.X.L. edited and wrote the paper. S.R.S., J.G.X., and Y.F.L. reviewed the paper.

## Supporting information



Supporting Information

Supplemental Video 1

Supplemental Video 2

Supplemental Video 3

Supplemental Video 4

## Data Availability

The data that support the findings of this study are available from the corresponding author upon reasonable request.
