# Supplementary material for: Cartilage‐Penetrating Framework Nucleic Acid Nanoparticles Ameliorate Osteoarthritis by Promoting Drug Delivery and Chondrocyte Uptake
Source: Adv Sci (Weinh). 2025 Apr 7;12(26):2502661. doi: 10.1002/advs.202502661 (PMC12245130; doi:10.1002/advs.202502661)
Supplement: Supplementary file 1 — Supporting Information [file ADVS-12-2502661-s003.docx]

**Supporting Information**

**Cartilage-penetrating framework nucleic acid nanoparticles ameliorate osteoarthritis by promoting drug delivery and chondrocyte uptake**

*Kui Huang, Qiumei Li, Huixuan Lin, Qian Shen, Yaping Wu, Taoran Tian, Chuan Ma, Sirong Shi***, Jingang Xiao***, and* *Yunfeng Lin**

**Table of Contents**

**S1. Supporting Figures**

S1.1 The structure of Pra-WL, Pra-WL-FITC and ginsenoside Rb1

S1.2 The characterization of tFNA-2WL and tFNA-2WL&Gin by TEM and AFM

S1.3 The synthesis and characterization of tFNA-nWL (n=1-4)

S1.4 The characterization of cartilage penetration of tFNA-nWL(n=1-4)

S1.5 The biodistribution and metabolism of tFNA-2WL in normal rat knee joints

S1.6 The biodistribution and metabolism of tFNA-2WL&Gin of OA knee joints in vivo

S1.7 The culture and characterization of chondrocytes

S1.8 The characterization of tFNA-2WL cytotoxicity and intracellular capacity

S1.9 The gait detection of rats in each treatment group

S1.10 The representative IVIS images and quantitative analysis of rat knee joints with skin removed of different groups after 1.5 h by injection of Cy5-CHP in vitro

S1.11 The representative 2D micro-CT images of knee joints and tibial epiphysis

S1.12 The representative images of H&E and Masson staining of cartilage from the rats after 4 weeks of different treatments

S1.13 The representative images of Col Ⅱ and MMP13 staining of cartilage from the rats after 4 weeks of different treatments.

S1.14 The representative images of Masson staining of synovium from the rats after 4 weeks of different treatments and the quantitative analysis of fluorescence intensity by Tunel staining in synovium

S1.15 The representative images of HE staining of heart, liver, spleen, lung, and kidney from the rats after 4 weeks of different treatments

**S2. Supporting Tables**

S2.1 The sequences of the oligonucleotides and peptides used in this study

S2.2 The list of Primer sequences used in this study

**S3. Supporting Videos**

S3.1 The light-sheet fluorescence microscopy imaging of the rat’s OA joint with intra-articular injection of Cy5-Gin

S3.2 The light-sheet fluorescence microscopy imaging of the rat’s OA joint with intra-articular injection of tFNA&Cy5-Gin.

S3.3 The light-sheet fluorescence microscopy imaging of the rat’s OA joint with intra-articular injection of tFNA-2WL&Cy5-Gin.

S3.4 The photographs of gait detection in rats after different treatments for 4 weeks

**Supplementary Figures**


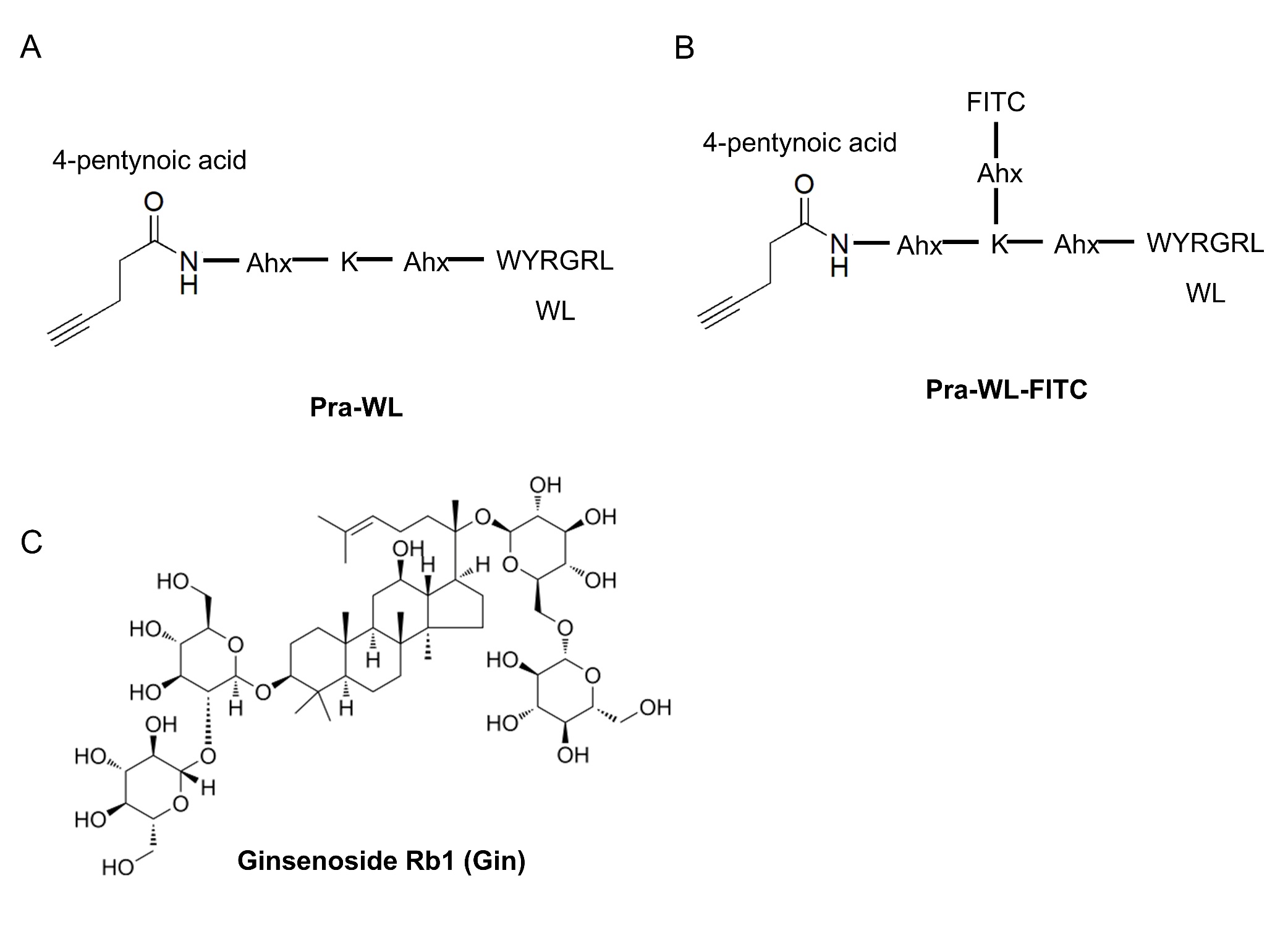


**Figure S1.** The structure of Pra-WL, Pra-WL-FITC and ginsenoside Rb1. (A, B) The structure of Pra-WL and Pra-WL-FITC. Ahx: 6-Aminohexanoic acid, Pra: 4-pentynoic acid. (C) The chemical structure of ginsenoside Rb1.


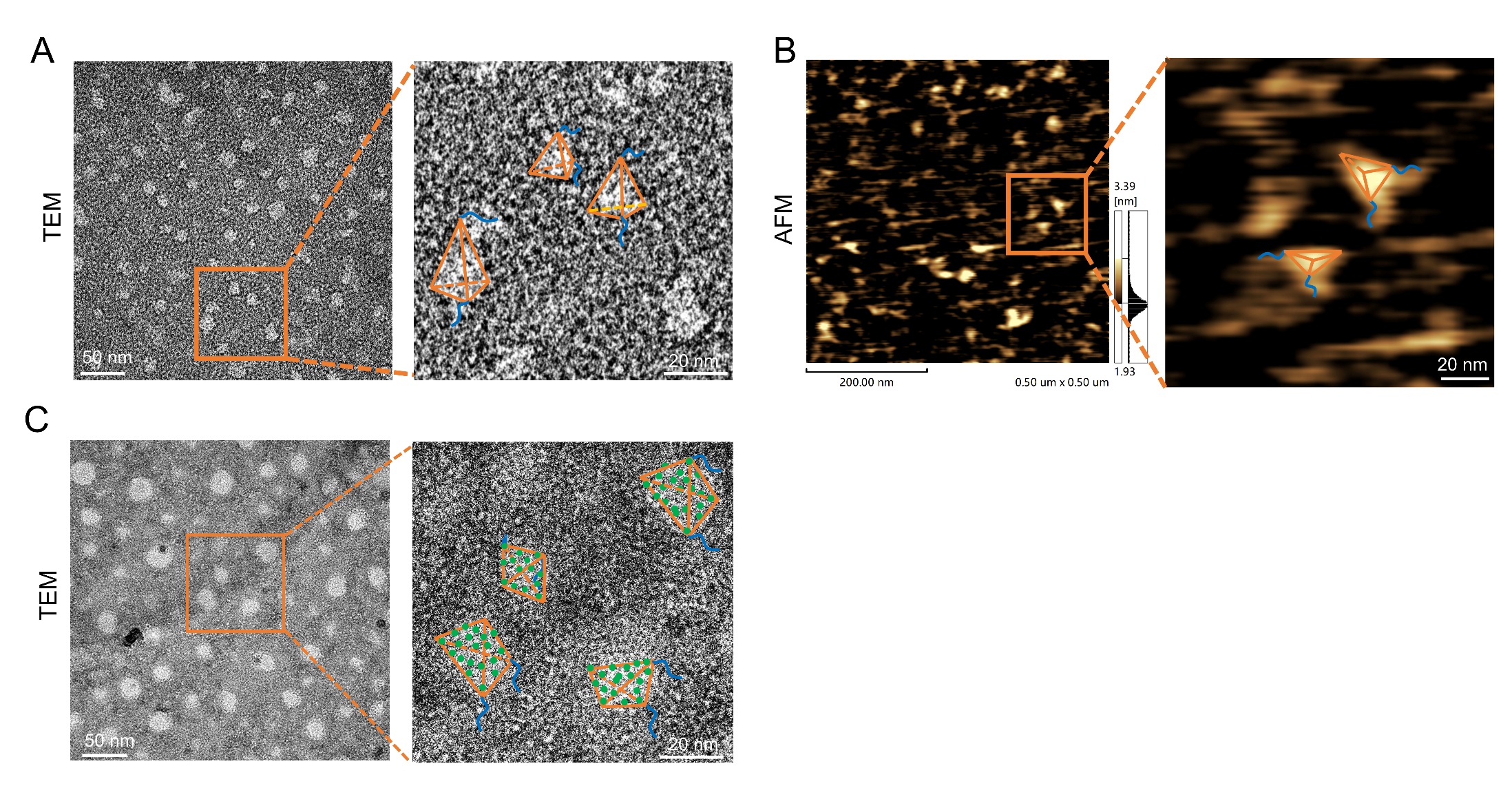


**Figure S2**. The characterization of tFNA-2WL and tFNA-2WL&Gin by TEM and AFM. (A) Representative TEM images of tFNA-2WL showing their molecule structure. Scale bar, 20 nm. (B) Representative AFM images of tFNA-2WL. Scale bar, 20 nm. (C) Representative TEM images of tFNA-2WL&Gin and its partial enlarged image. Scale bar, 50nm and 20 nm.


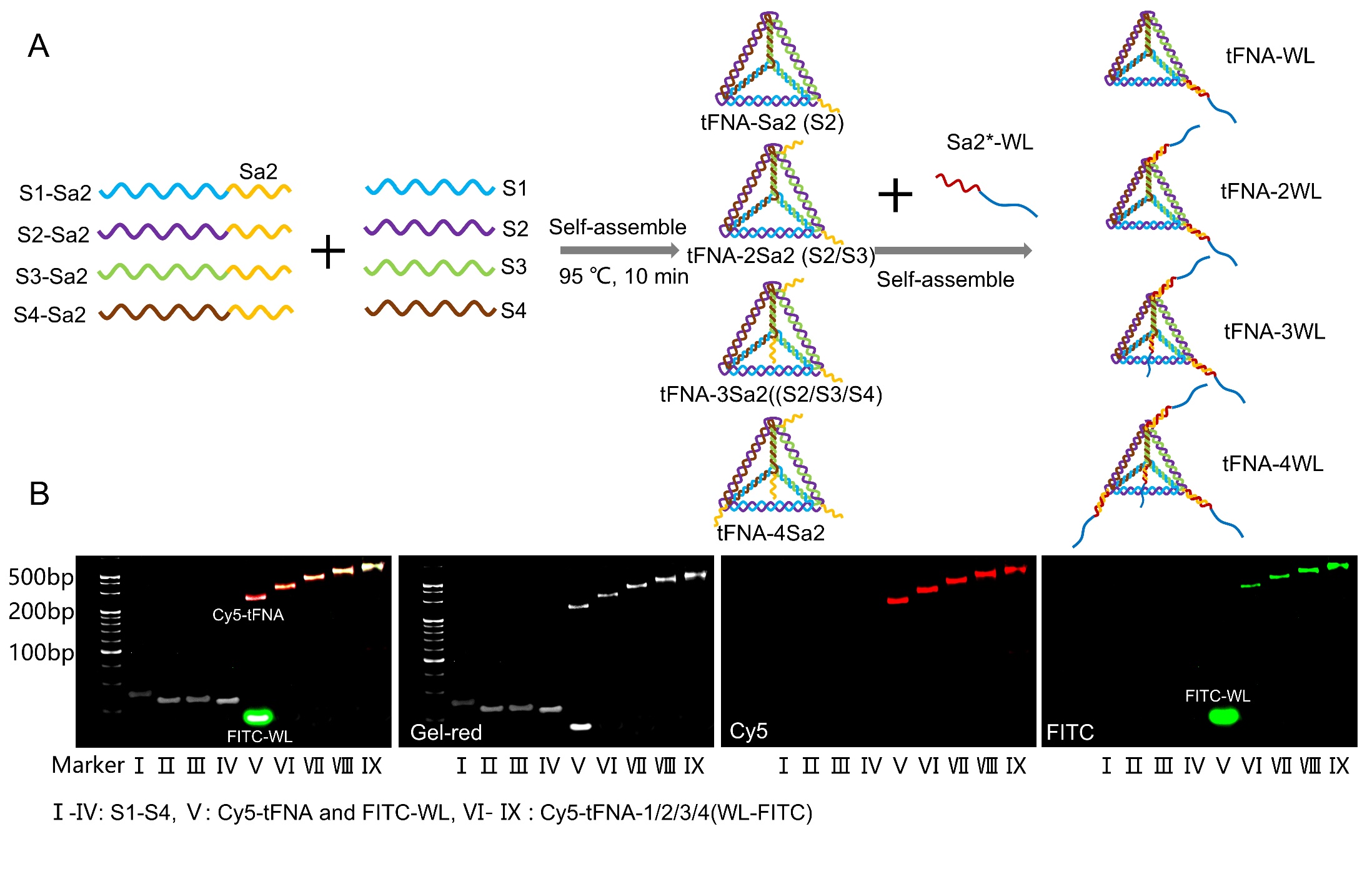


**Figure S3.** The synthesis and characterization of tFNA-nWL (n=1-4). (A) Schematic illustration of the synthesis of tFNA-nWL (n=1-4). (B) Representative PAGE images of tFNA-nWL (n=1-4), the results showed that tFNA-nWL’s molecular weight gradual increased with the increase of n, and the fluorescence of Cy5-tFNA and WL-FITC achieved good co-localization (lane Ⅵ-Ⅸ), and Cy5-tFNA and WL-FITC cannot bind without modification of Sa2 and Sa2*(lane Ⅴ). (tFNA were labeled with Cy5 (red) and WL were labeled FITC).


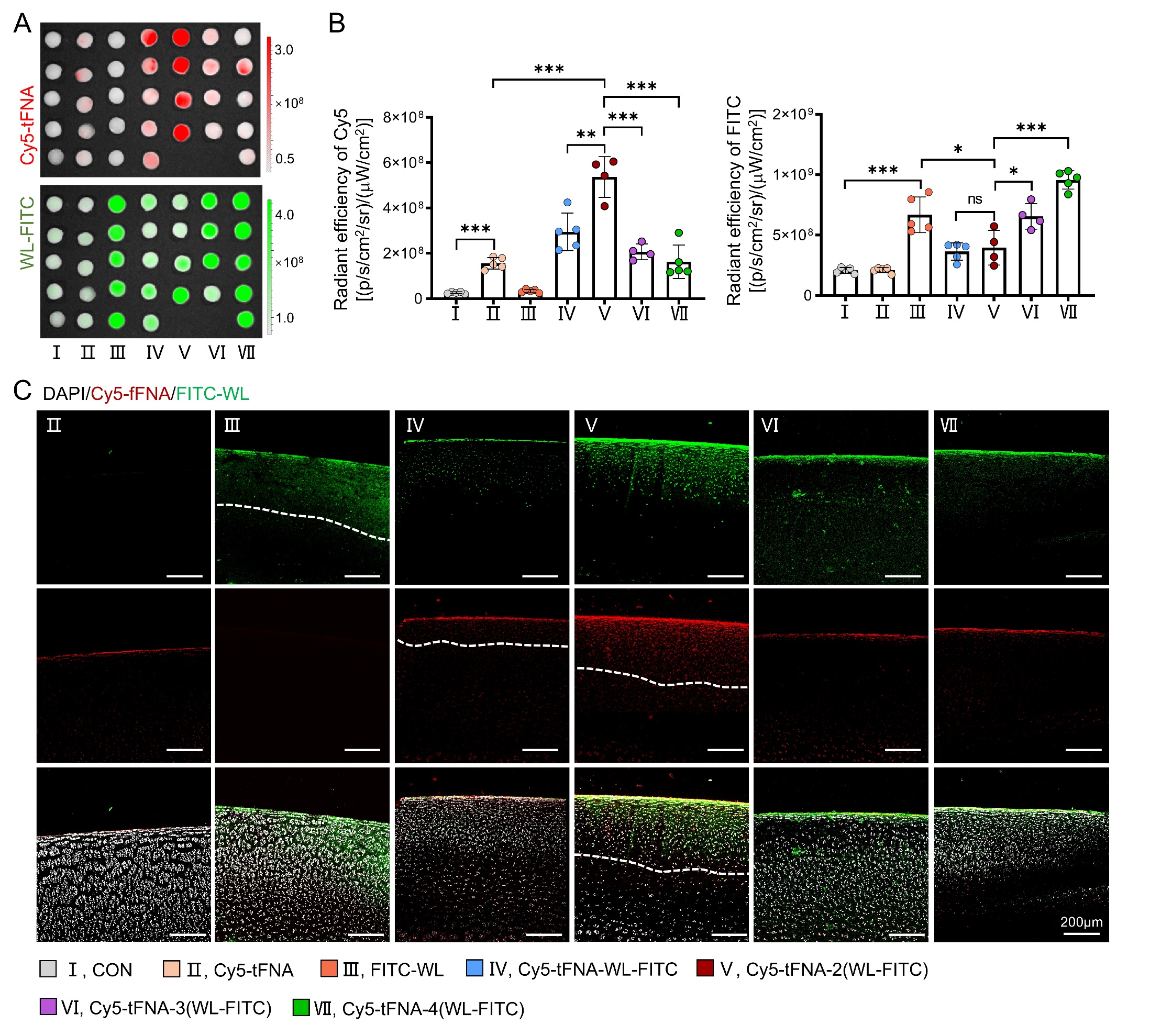


**Figure S4**. The characterization of cartilage penetration of tFNA-nWL(n=1-4). (A and B) Fluorescence images and quantitative analysis of cartilage explants incubated with CON (PBS), tFNA (Cy5-tFNA), WL (WL-FITC) and tFNA-nWL [Cy5-tFNA-n(WL-FITC), n=1-4] for 12h using IVIS in FITC and Cy5 fluorescence channels, respectively. *n=4 or 5*. (C) Representative cross-sectional scanning images of cartilages cartilage explants of different groups above by confocal microscopy, those results indicated that tFNA-2WL(Ⅴ) owns the strongest cartilage penetration ability among the tFNA-nWL (n=1-4) (shown by the white dotted line). Scale bar: 200 μm. All data are presented as the mean ± SD. Statistical analysis was analyzed by one-way ANOVA with Tukey’s multiple comparisons test for (B). **P* < 0.05, ***P* < 0.01, ****P* < 0.001, *****P* < 0.0001.


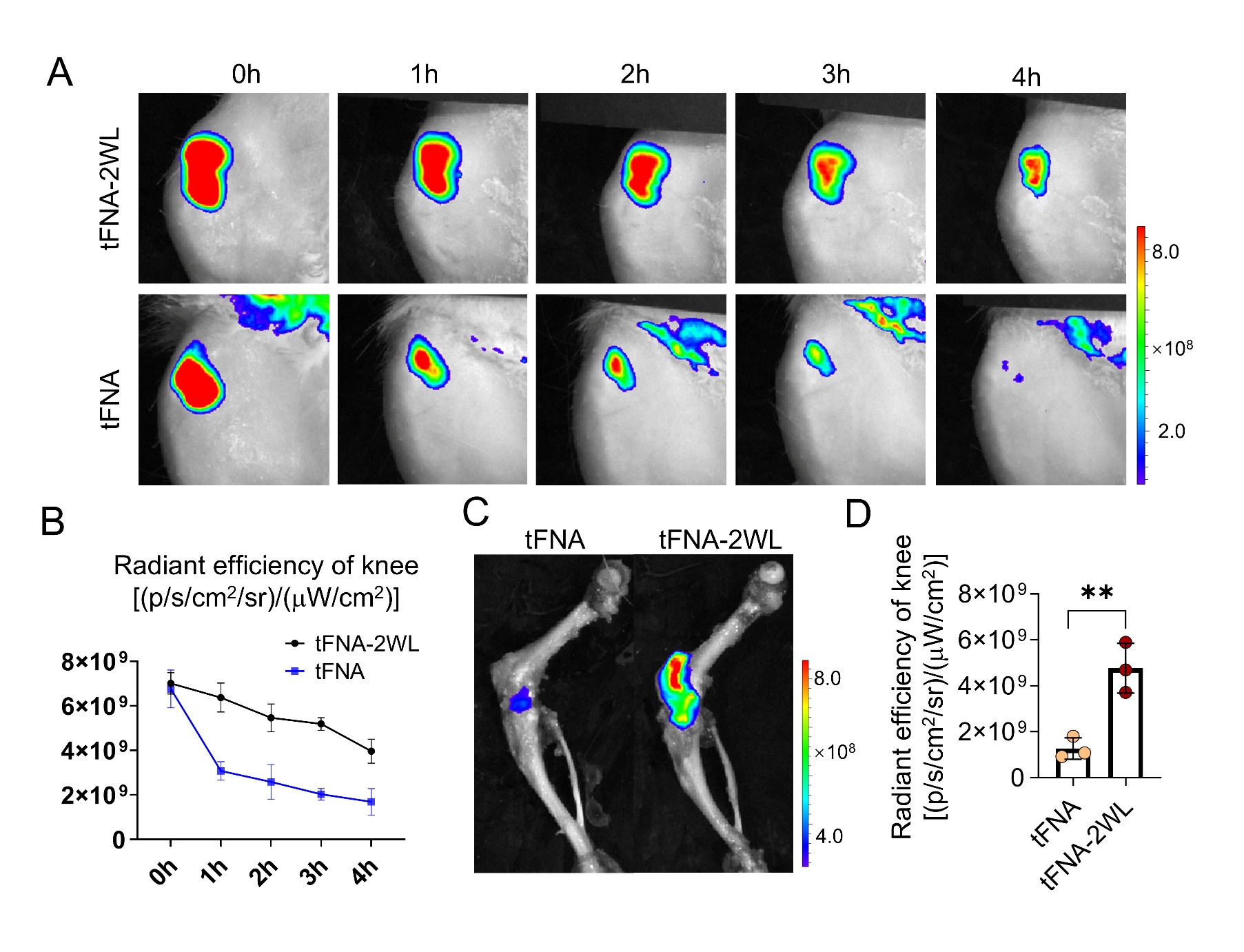


**Figure S5.** The biodistribution and metabolism of tFNA-2WL in normal rat knee joints. (A and B) Representative IVIS images and quantitative analysis of normal rat knee joints (8 weeks old) over 4h after injection of Cy5-tFNA, and Cy5-tFNA-2(WL-FITC) in vivo. *n=3*. (C and D) Representative IVIS images and quantitative analysis of knee joints with skin removed 4 hours after Cy5-tFNA and Cy5-tFNA-2(WL-FITC) injection *in vitro*. *n=3*. All data are presented as the mean ± SD. Statistical analysis was analyzed by one-way ANOVA with Tukey’s multiple comparisons test for (D). ***P* < 0.01.


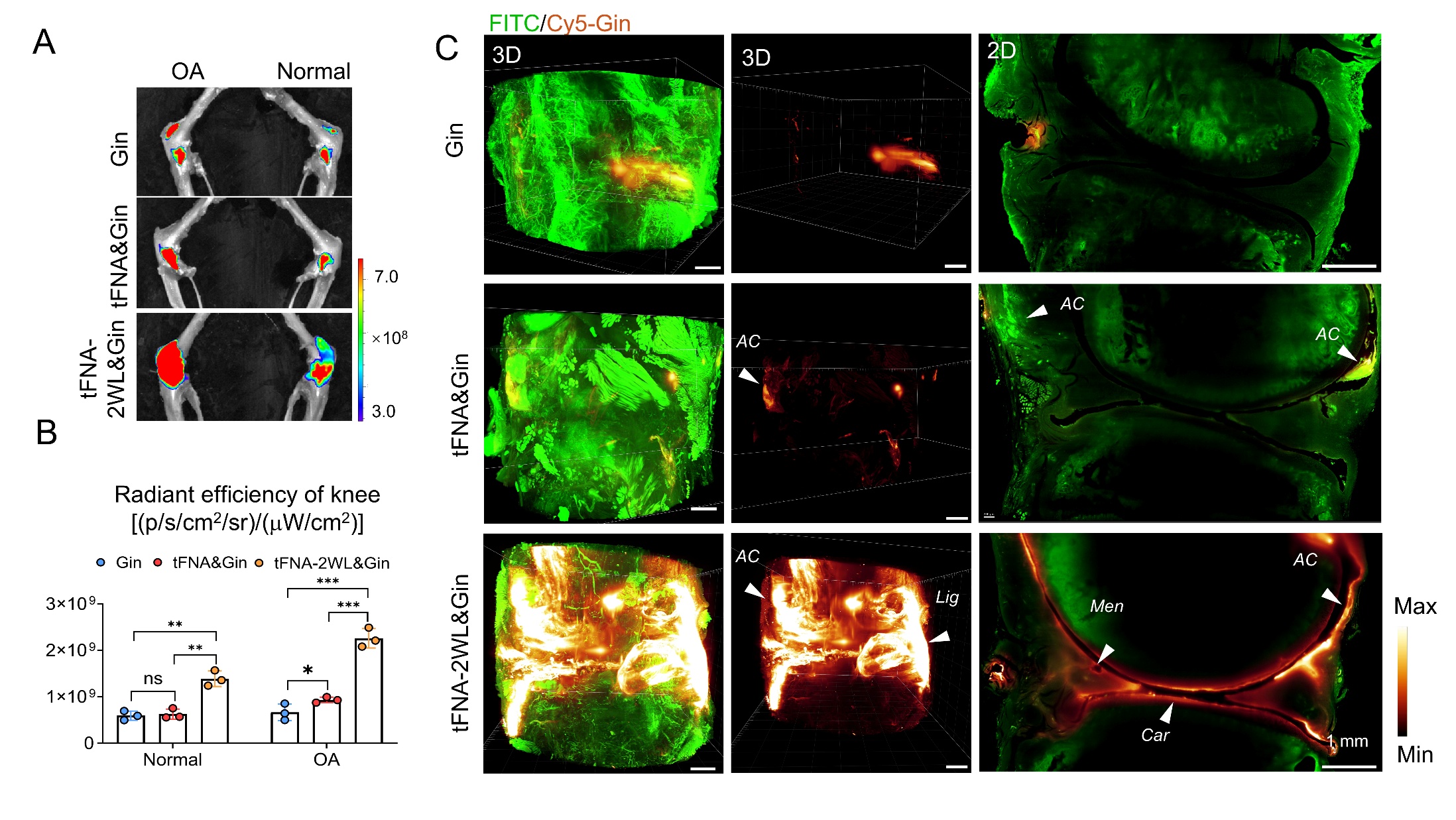


**Figure S6.** The biodistribution and metabolism of tFNA-2WL&Gin of OA knee joints in vivo. (A and B) Representative IVIS images and quantitative analysis of OA and normal knee joints with skin removed 7 hours after Cy5-Gin, tFNA&Cy5-Gin and tFNA-2(WL-FITC)&Cy5-Gin injection in vitro. n=3. (C) Representative 2D light-sheet microscopy images and three-dimensional (3D) reconstruction images of the rat’s OA joint with intra-articular injection of Cy5-Gin, tFNA&Cy5-Gin and tFNA-2WL-FITC&Cy5-Gin, tissue clarity and three-dimensional fluorescence imaging, the results showed that tFNA-2WL&Cy5-Gin could be retained in cartilage, meniscus, joint ligaments and synovium, while only tFNA&Cy5-Gin of the other two groups was only slightly enriched in the synovium (shown by white arrows). Note: gold, Cy5-Gin; green, tissue autofluorescence. *AC*: articular capsule, *Lig*: ligment, *Men*: meniscus, *Car*: cartilage. Scale bars, 1mm. Data are shown as the mean ± SD. Statistical analysis was analyzed by two-way ANOVA with Tukey’s multiple comparisons test for (B). **P* < 0.05, ***P* < 0.01, ****P* < 0.001.


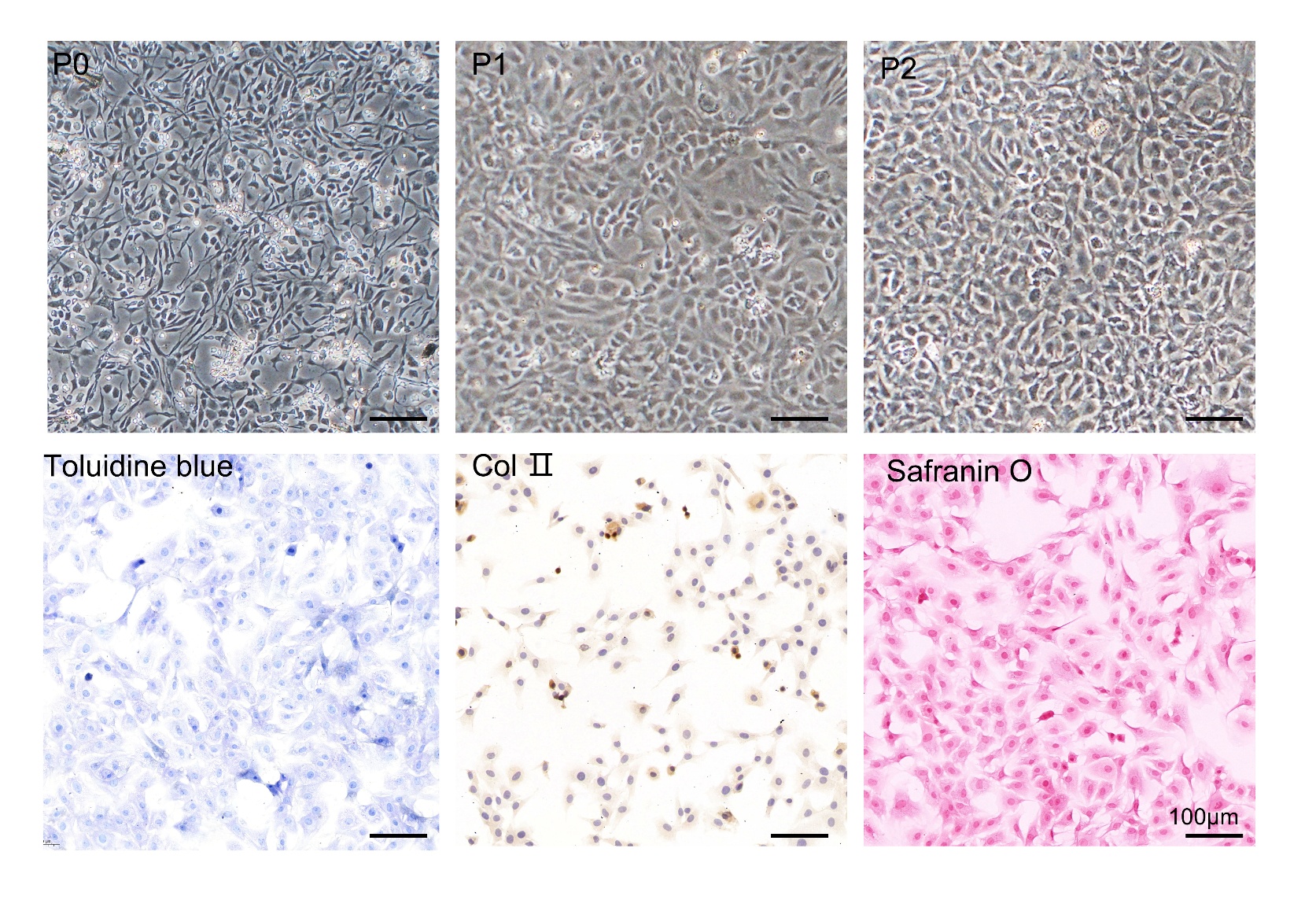


**Figure S7.** The culture and characterization of chondrocytes. Representative light microscopy images of chondrocytes of generation P0, P1, P2, and P1 chondrocytes stained with Toluidine blue, Col Ⅱ, and SO&FG.


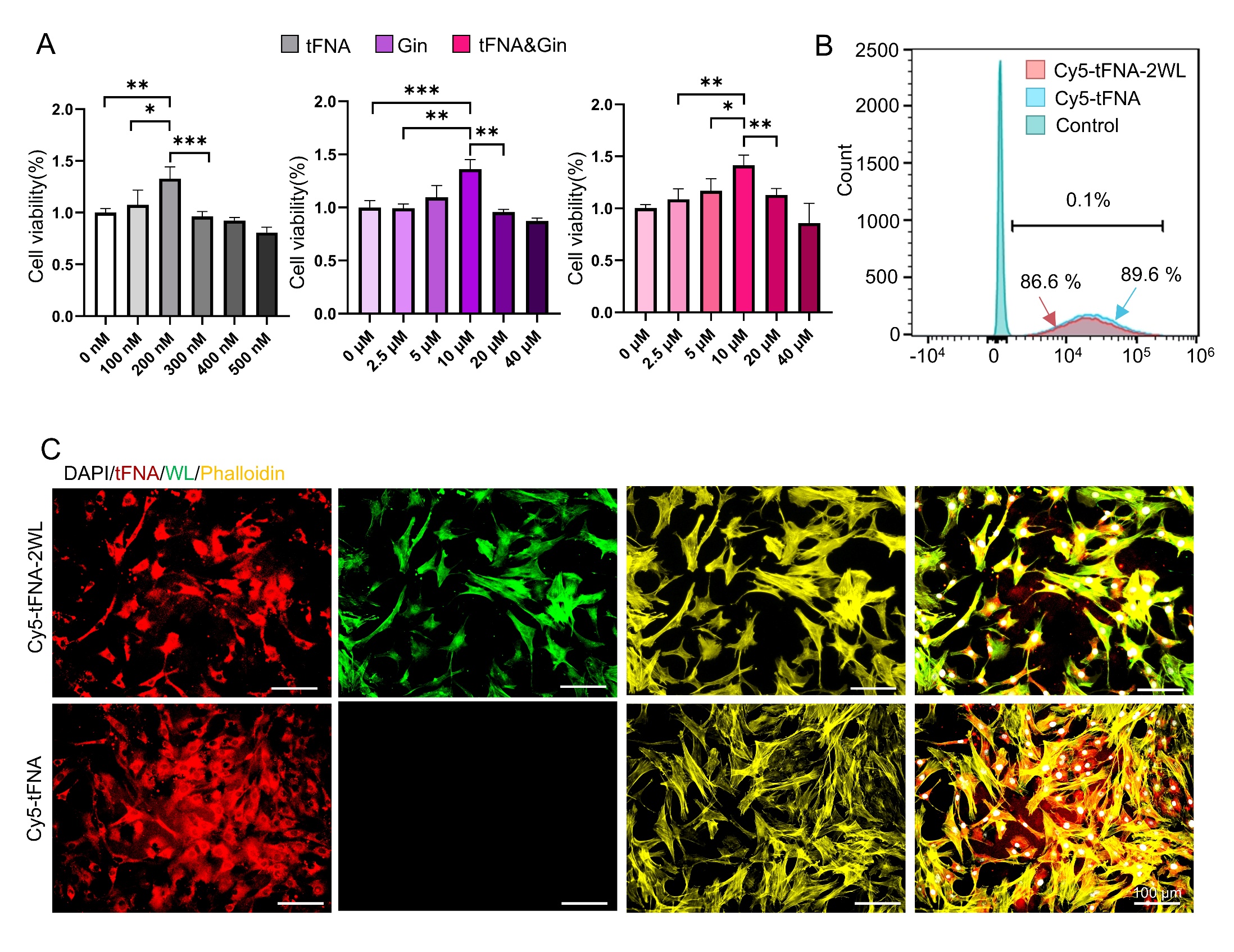


**Figure S8.** The characterization of tFNA-2WL cytotoxicity and intracellular capacity. (A) Cytotoxicity of nanomedicine systems of tFNA, Gin and tFNA&Gin, measured by CCK8 kit after co-incubation of chondrocytes with different concentrations (tFNA: 0,100, 200, 300, 400, 500 nM; Gin: 0, 2.5, 5, 10, 20, 40 μM) for 12h. (B) Evaluation of cell entry efficiency of Cy5-tFNA and Cy5-tFNA-2(WL-FITC) measured by flow cytometry. (C) Representative confocal fluorescence images of chondrocytes co-cultureed with Cy5-tFNA, Cy5-tFNA-2(WL-FITC) for 6h. Scale bars: 100μm. Data are presented as the mean ± SD. Statistical analysis was analyzed by two-way ANOVA with Tukey’s multiple comparisons test for (A). **P* < 0.05, ***P* < 0.01, ****P* < 0.001.


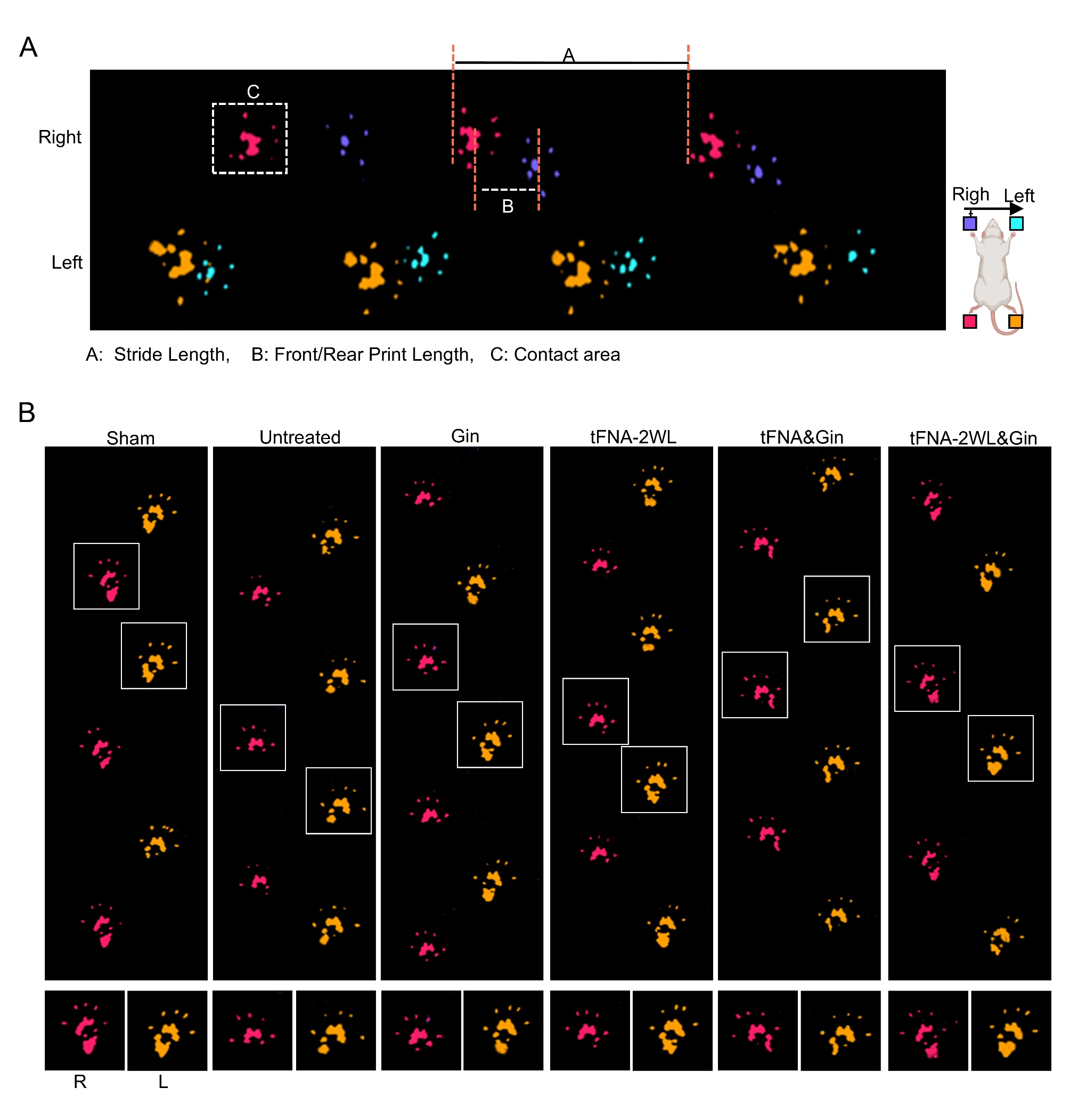


**Figure S9.** The gait detection of rats in each treatment group. (A) Schematic illustration of measuring indicators for footprints analysis. (B) Photographs of the rats’ hind limb footprints after different treatments for 4 weeks showing the difference in the maximum contact area of ​​the right hind limbs of rats in each group. Left hind foot, yellow; Right hind foot, red. R: right, L: left.


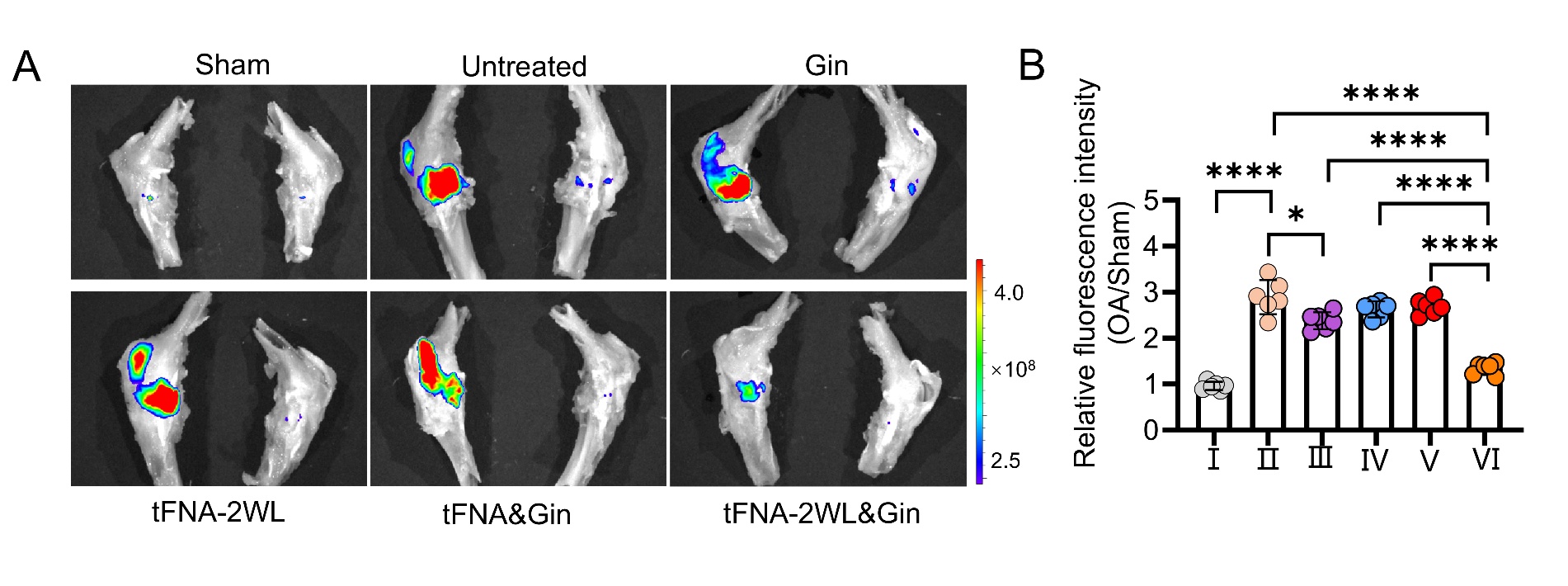


**Figure S10.** The representative IVIS images (A) and quantitative analysis (B) of rat knee joints with skin removed of different groups after 1.5 h by injection of Cy5-CHP in vitro. *n=6*. All data are presented as the mean ± SD. Statistical analysis was analyzed by two-way ANOVA with Tukey’s multiple comparisons test for (B). **P* < 0.05, ***P* < 0.01, ****P* < 0.001.


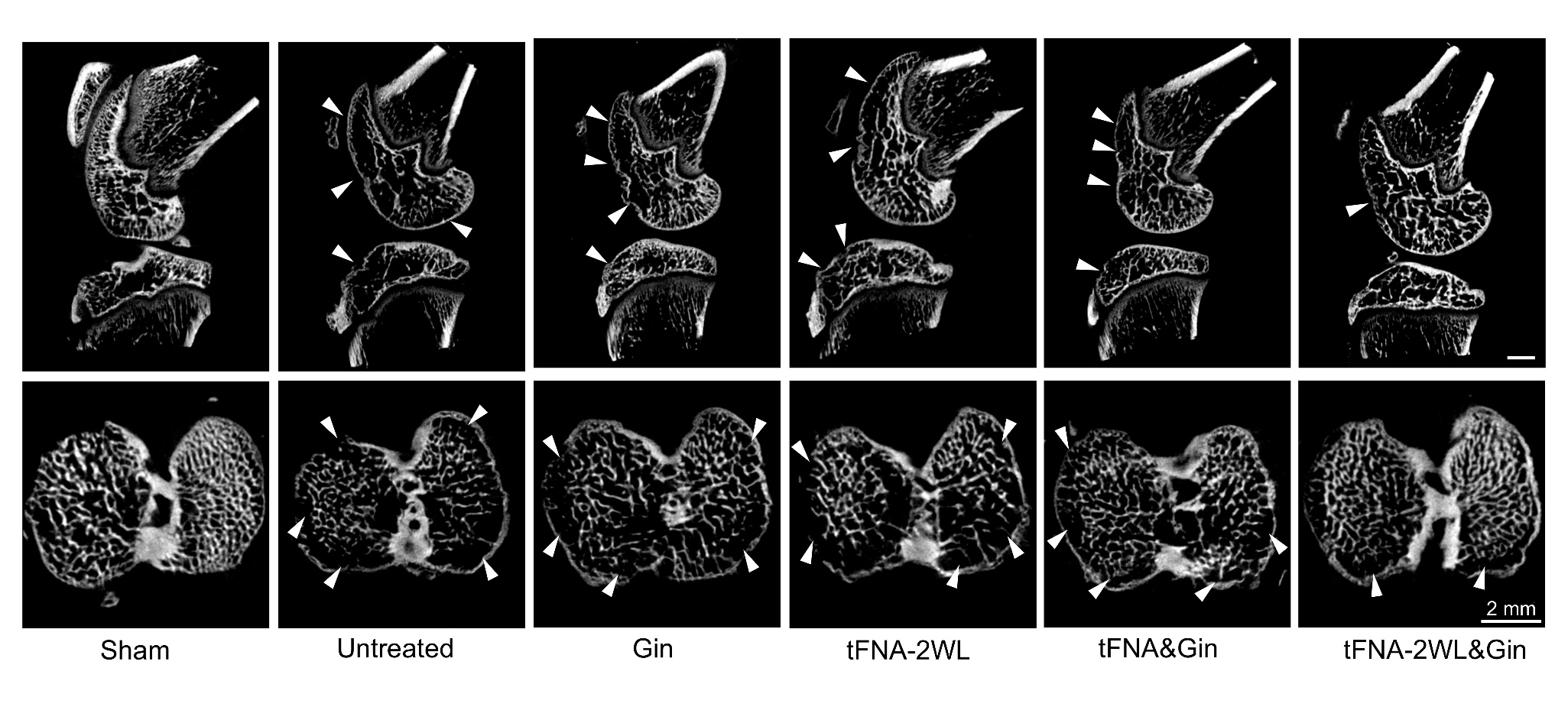


**Figure S11.** The representative 2D micro-CT images of knee joints (the first row, coronal plane) and tibial epiphysis (the second row, horizontal plane) showing the occurrence of osteophytes, bone defects and bone resorption in all groups (shown by white arrows). Scale bars: 2 mm.


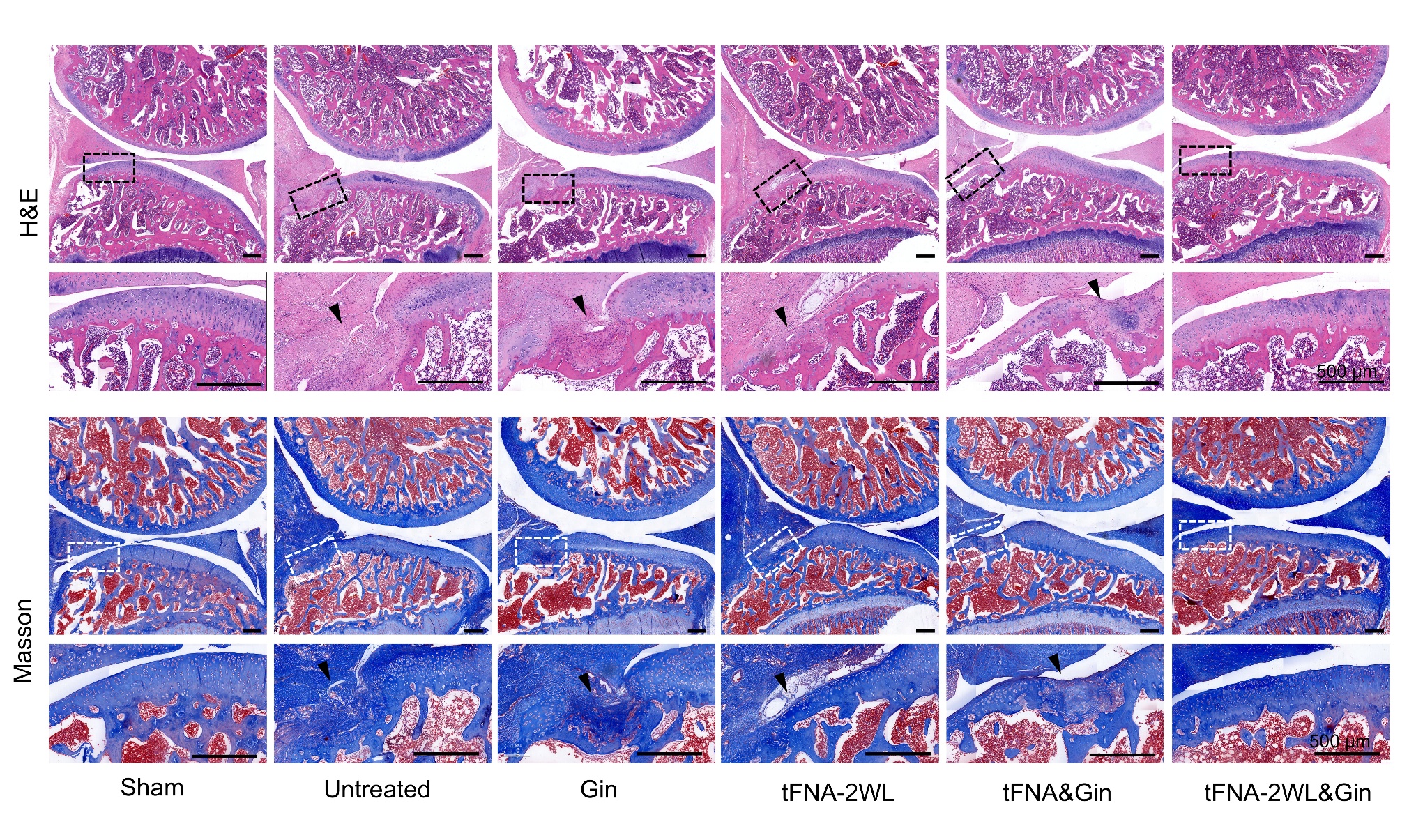


**Figure S12.** The representative images of H&E and Masson staining of cartilage from the rats after 4 weeks of different treatments. Scale bars, 500μm.


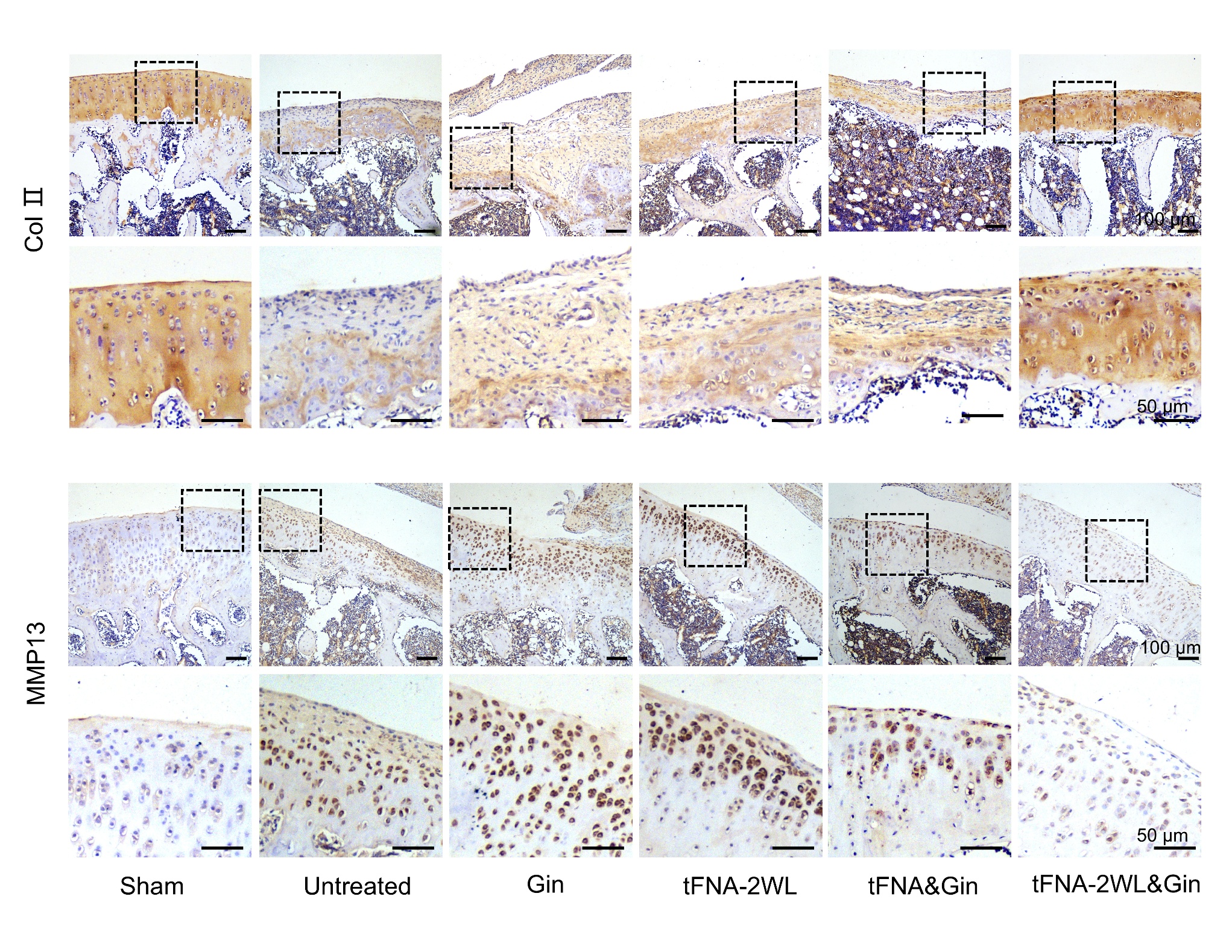


**Figure S13.** The representative images of Col Ⅱ and MMP13 staining of cartilage from the rats after 4 weeks of different treatments. Scale bars, 100 μm or 50 μm.


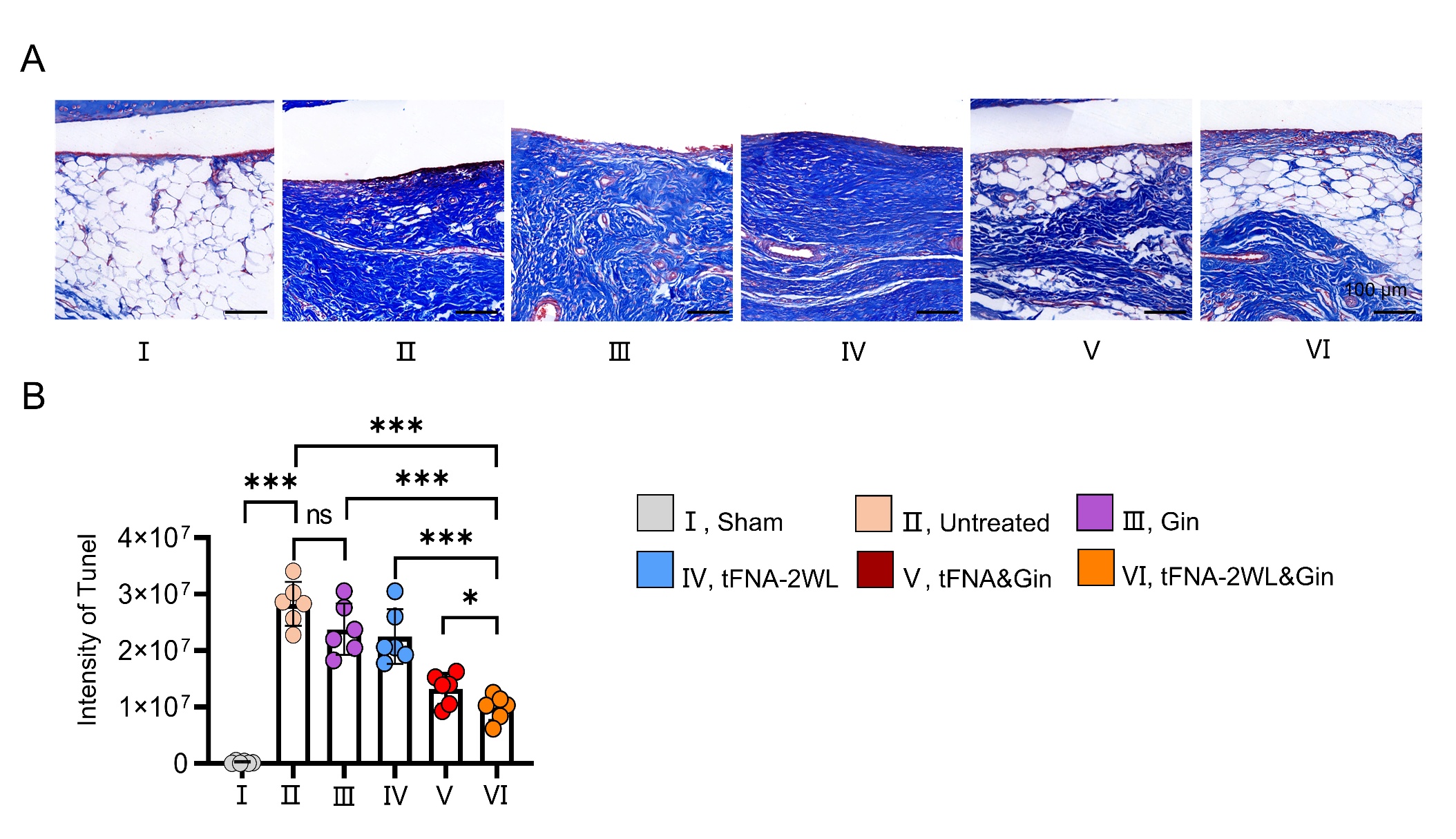


**Figure S14.** (A) The representative images of Masson staining of synovium from the rats after 4 weeks of different treatments. Scale bars, 100 μm. (B) Quantitative analysis of fluorescence intensity by Tunel staining in synovium. *n=6*. Data are shown as the mean ± SD and statistical analysis was performed by one-way ANOVA with Tukey’s multiple comparisons test for (B). **P* < 0.05, ****P* < 0.001.


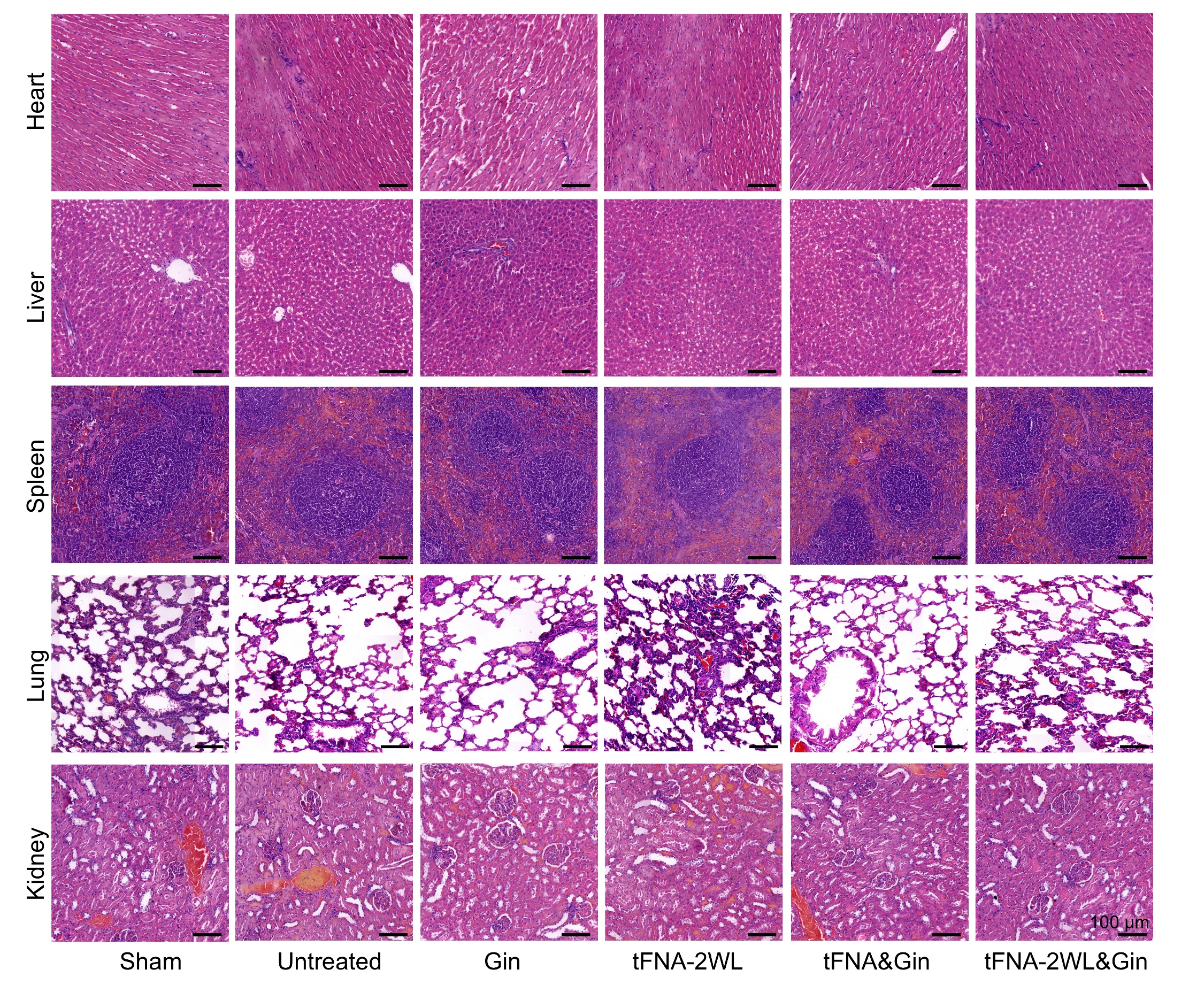


**Figure S15.** The representative images of HE staining of heart, liver, spleen, lung, and kidney from the rats after 4 weeks of different treatments, the results indicated no toxicity of all nanomedicines *in vivo***.** Scale bar: 100 μm.

**Table S1.** **Sequences of the oligonucleotides and peptides used in this study**

| **Name** | **Sequences (5’-3’)** |
| --- | --- |
| S1 | ATT TAT CAC CCG CCA TAG TAG ACG TAT CAC CAG GCA GTT GAG ACG AAC ATT CCT AAG TCT GAA |
| S2 | ACA TGC GAG GGT CCA ATA CCG ACG ATT ACA GCT TGC TAC ACG ATT CAG ACT TAG GAA TGT TCG |
| S3 | ACT ACT ATG GCG GGT GAT AAA ACG TGT AGC AAG CTG TAA TCG ACG GGA AGA GCA TGC CCA TCC |
| S4 | ACG GTA TTG GAC CCT CGC ATG ACT CAA CTG CCT GGT GAT ACG AGG ATG GGC ATG CTC TTC CCG |
| Cy5-S1 | Cy5-ATT TAT CAC CCG CCA TAG TAG ACG TAT CAC CAG GCA GTT GAG ACG AAC ATT CCT AAG TCT GAA |
| S1-Sa2 | **TAC CAC CTA CAT CAC TTT TG** ATT TAT CAC CCG CCA TAG TAG ACG TAT CAC CAG GCA GTT GAG ACG AAC ATT CCT AAG TCT GAA |
| S2-Sa2 | **TAC CAC CTA CAT CAC TTT TG** ACA TGC GAG GGT CCA ATA CCG ACG ATT ACA GCT TGC TAC ACG ATT CAG ACT TAG GAA TGT TCG |
| S3-Sa2 | **TAC CAC CTA CAT CAC TTT TG** ACT ACT ATG GCG GGT GAT AAA ACG TGT AGC AAG CTG TAA TCG ACG GGA AGA GCA TGC CCA TCC |
| S4-Sa2 | **TAC CAC CTA CAT CAC TTT TG** ACG GTA TTG GAC CCT CGC ATG ACT CAA CTG CCT GGT GAT ACG AGG ATG GGC ATG CTC TTC CCG |
| Sa2*-N3 | GTG ATG TAG GTG GTA-N3 |
| Pra-WL-FITC | Ac-Pra-Ahx-Lys(FITC-Ahx)-Ahx-Trp-Tyr-Arg-Gly-Arg-Leu-NH_2_ |
| Pra-WL | Ac-Pra-Ahx-Lys-Ahx-Trp-Tyr-Arg-Gly-Arg-Leu-NH_2_ |
| Cy5-CHP | Cy5-Ahx-GfOGfOGfOGfOGfOGfOGfOGfOGfO |
|  |  |

**Table S2. List of Primer sequences used in this study**

| **Gene** | **Primer** | **Sequences** |
| --- | --- | --- |
| GAPDH | Forward | GACATGCCGCCTGGAGAAAC |
|  | Reverse | AGCCCAGGATGCCCTTTAGT |
| ACAN | Forward | GATCTCAGTGGGCAACCTTC |
|  | Reverse | TCCACAAACGTAATGCCAGA |
| Col-Ⅱ | Forward | GGAGCAGCAAGAGCAAGGAGAAG |
|  | Reverse | TCAGTGGACAGTAGACGGAGGAAAG |
| ADAMTS5 | Forward | GTCCAAATGCACTTCAGCCACGAT |
|  | Reverse | AATGTCAAGTTGCACTGCTGGGTG |
| MMP13 | Forward | GCGGGAATCCTGAAGGAGAATGC |
|  | Reverse | TCAAGTTTGCCAGTCACCTCTAAGC |

**Supplementary Videos**

**Video S1.** Light-sheet fluorescence microscopy imaging of the rat’s OA joint with intra-articular injection of Cy5-Gin. The results indicated that Cy5-Gin is cleared rapidly, with essentially no retention in OA joints. Cy5-Gin was injected into the knee joints of rats 7 h before joints collection. Gold, Cy5-Gin; Green, tissue autofluorescence. Scale bars, 1mm.

**Video S2.** Light-sheet fluorescence microscopy imaging of the rat’s OA joint with intra-articular injection of tFNA&Cy5-Gin. The results showed that tFNA&Cy5-Gin was only slightly retained in the synovium, but hardly retained in cartilage and meniscus. tFNA&Cy5-Gin was also injected into the knee joints of rats 7 h before joint tissue collection. Gold, tFNA&Cy5-Gin; Green, tissue autofluorescence. Scale bars, 1mm.

**Video S3.** Light-sheet fluorescence microscopy imaging of the rat’s OA joint with intra-articular injection of tFNA-2WL&Cy5-Gin. The results showed that tFNA-2WL&Cy5-Gin could be abundantly retained in cartilage, meniscus, joint ligaments, and synovium, which in turn prolongs the retention time of Gin. Gold, tFNA-2WL&Cy5-Gin; Green, tissue autofluorescence. Scale bars, 1mm.

**Video S4.** Photographs of gait detection in rats after different treatments for 4 weeks showe that tFNA-2WL&Gin treatment significantly increased the walking speed of OA rats (0.5× speed).
